# Electrochemical CO_2_ Reduction on Janus Dual‐Atom Catalysts: Critical Role of Oxygen Coordination and an Effective Descriptor

**DOI:** 10.1002/advs.202507849

**Published:** 2025-07-08

**Authors:** Hongling Liu, Zhichao Yu, Jia Zhao, Weng Fai Ip, Sen Lin, Hui Pan

**Affiliations:** ^1^ Institute of Applied Physics and Materials Engineering University of Macau Macao SAR 999708 P. R. China; ^2^ Department of Physics and Chemistry Faculty of Science and Technology University of Macau Macao SAR 999078 P. R. China; ^3^ State Key Laboratory of Photocatalysis on Energy and Environment College of Chemistry Fuzhou University Fuzhou 350116 P. R. China

**Keywords:** CO_2_ electroreduction, density functional theory, descriptor, Janus dual‐atom catalysts, methanol, methane

## Abstract

Dual‐atom catalysts (DACs) embedded in nitrogen‐doped graphene have been widely studied for electrochemical CO_2_ reduction (CO_2_R), primarily yielding CO. However, achieving selectivity for C1 hydrocarbons remains challenging. Here, 32 Janus DACs (J‐M'M) are designed and investigated for CO_2_R using density functional theory (DFT) calculations, identifying 13 capable of producing methanol and methane. Notably, J‐FeCo and J‐CoNi exhibit favorable limiting potentials (−0.38 and −0.45 V vs. RHE) for CH_3_OH and CH_4_ production, respectively, based on constant‐potential calculations. Compared to normal DACs (N‐M'M), Janus DACs demonstrate enhanced initial CO_2_ hydrogenation and stronger CO adsorption. Oxygen coordination in J‐FeCo and J‐CoNi induces a downshift/upshift of majority‐/minority‐spin energy levels of d_z2_, d_yz_, and d_xz_ orbitals toward the Fermi level relative to N‐FeCo and N‐CoNi, strengthening the bonding state and weakening the antibonding state, thereby improving CO adsorption. Furthermore, an effective descriptor based on atomic features is identified to evaluate *CO binding strength. This work highlights the critical role of partial oxygen coordination in DACs for C1 hydrocarbons production and proposes a robust descriptor to guide the design of related catalysts.

## Graphical abstract

Janus dual‐atom catalysts (DACs) with N_3_O_3_ hybrid coordination are designed with different combination of metal sites. Compared to normal DACs, most Janus DACs favor CH_3_OH or CH_4_ production over CO, driven by enhanced initial CO_2_ hydrogenation and stronger CO adsorption.

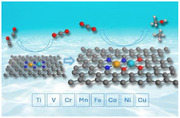

## 1 Introduction

The excessive consumption of fossil fuels and subsequent emissions of carbon dioxide (CO_2_) have resulted in a series of environmental issues and energy crisis. Electrochemical CO_2_ reduction (CO_2_R) to value‐added chemicals has been recognized as an appealing strategy for alleviating the greenhouse effect and achieving carbon‐neutral circulation.^[^ ^1^, ^2^, ^3^ ^]^ Extensive efforts have been devoted to developing heterogeneous electrocatalysts for efficient and selective CO_2_R, including metals,^[^ ^4^ ^]^ metal oxides,^[^ ^5^ ^]^ metal alloys,^[^ ^6^ ^]^ and carbon‐based materials.^[^ ^7^, ^8^ ^]^ Atomically dispersed single‐atom catalysts (SACs) have flourished in the field of electrochemical CO_2_R in recent years owing to the maximal utilization of active atoms, well‐defined structure, and unique electronic properties.^[^ ^9^, ^10^ ^]^ However, some typical M‐N_4_‐C (M is metal atom, such as Fe, Co, Ni, and Cu) SACs show unsuitable adsorption strength of intermediates, such as *COOH and *CO. For example, Ni and Cu SACs demonstrate kinetic limitations in the first proton‐electron transfer step. In contrast, Fe and Co SACs exhibit low onset potential, but struggle with *CO desorption.^[^ ^11^, ^12^, ^13^ ^]^ Generally, the adsorption configuration of intermediate is determined by the individual active sites, which causes a linear scaling relationship between key intermediates. It is challenging to simultaneously optimize and tune the adsorption of different intermediates at the single active site. In addition, although some M‐N_4_‐C SACs attain the product selectivity up to ≈100%, most are generally limited to two‐electron reduction product CO,^[^ ^12^, ^14^ ^]^ which can be attributed to the relatively weak bonding strength of *COOH/*CO and strong C─O bond in CO.^[^ ^15^ ^]^

Dual‐atom catalysts (DACs) as a rising star not only inherit the merits of SACs, but also could optimize the electronic properties via homo‐/hetero‐nuclear interatomic synergy, holding potential in breaking the linear scaling relation.^[^ ^16^, ^17^ ^]^ For instance, Fe_2_−N−C DAC could improve the overly strong adsorption strength of *CO in Fe−N−C SAC via orbital coupling between the dual Fe sites.^[^ ^18^ ^]^ Similarly, CoCu DAC could make up for the shortcomings of Co/Cu SACs and maintain the advantages of both.^[^ ^19^ ^]^ Different combinations of metals for DACs, such as FeCu,^[^ ^20^ ^]^ NiCu,^[^ ^21^ ^]^ and FeNi,^[^ ^13^ ^]^ have been developed and show superior CO_2_R performance to those of SACs. However, CO is still identified as the main product on almost all homonuclear and heteronuclear DACs.^[^ ^21^, ^22^, ^23^, ^24^, ^25^ ^]^ The reduction of CO to hydrocarbons on some DACs with pyridine N atom coordination was demonstrated to be unfavorable compared to CO desorption.^[^ ^23^ ^]^

Coordination environment engineering is widely applied for electrocatalysts including SACs and DACs to optimize catalytic performance in oxygen reduction/evolution reaction (ORR/OER),^[^ ^26^, ^27^ ^]^ CO_2_R,^[^ ^28^, ^29^, ^30^ ^]^ electrocatalytic nitrate reduction (NO_3_R),^[^ ^31^ ^]^ and nitrogen reduction reaction (NRR).^[^ ^32^ ^]^ Specifically, some non‐metal elements besides nitrogen (e.g. O, B, S, P) could be incorporated to further diversify the coordination environment, thus tuning the catalytic activity and selectivity.^[^ ^33^, ^34^, ^35^ ^]^ For example, CuN_2_O_2_ SAC was synthesized for electrochemical converting CO_2_ to CH_4_ with high Faraday efficiency and selectivity.^[^ ^36^ ^]^ Interestingly, the CoO*_n_* N_4–_ *_n_* exhibited significantly lower adsorption energies for *CO and *COOH than CoC*_n_* N_4–_ *_n_*,^[^ ^37^ ^]^ indicating that the suitable incorporation of the oxygen atom can strengthen the bonding of *COOH and *CO. The strengthened CO adsorption holds potential to promote CO_2_R process for the further reduction of CO to hydrocarbons. As such, modifying the coordination environment of DACs extends the synergistic effect, which benefits the multistep catalytic reactions. Although significant efforts have been made, tuning DACs with partial oxygen coordination environment to regulate the selectivity for hydrocarbon products is still highly pursued in electrochemical CO_2_R.

It has been reported that metal‐based Janus nanostructures integrated two discrepant components could perform more synergetic functions in CO_2_R.^[^ ^38^, ^39^ ^]^ Recently, Tang et al. successfully synthesized some Janus DACs (FeCo–N_3_O_3_@C, FeCu–N_3_O_3_@C, FeNi–N_3_O_3_@C) with two coordination environment for electrocatalytic OER/ORR.^[^ ^27^ ^]^ Building on the framework proposed by Tang et al., we expand the family of Janus dual‐atom catalysts (DACs) and evaluate their CO_2_ reduction (CO_2_R) performance using density functional theory (DFT) calculations. We investigated 32 Janus DACs (J‐M'M), with M’ (Fe, Co, Ni, Cu) coordinated to nitrogen and M (Ti, V, Cr, Mn, Fe, Co, Ni, Cu) coordinated to oxygen, assessing their stability, selectivity, and activity sequentially. Among these, J‐FeCo and J‐CoNi exhibit exceptional CO_2_R activity, producing CH_3_OH and CH_4_ with favorable limiting potentials of −0.38 and −0.45 V vs. RHE, respectively, under operational conditions. Compared to normal DACs (N‐M'M), most Janus DACs favor CH_3_OH or CH_4_ production over CO, driven by stronger *COOH and *CO binding. Notably, the linear scaling relations for key adsorbates (*COOH, *CO, and *CHO) are disrupted in Janus DACs. Leveraging intrinsic atomic properties, we identified an effective descriptor to predict *CO adsorption energy, offering a robust tool for designing Janus DACs for enhanced CO_2_R performance.

## 2 Results and Discussion

### 2.1 Geometric Structure and Stability of Janus DACs

**Figure 1:**
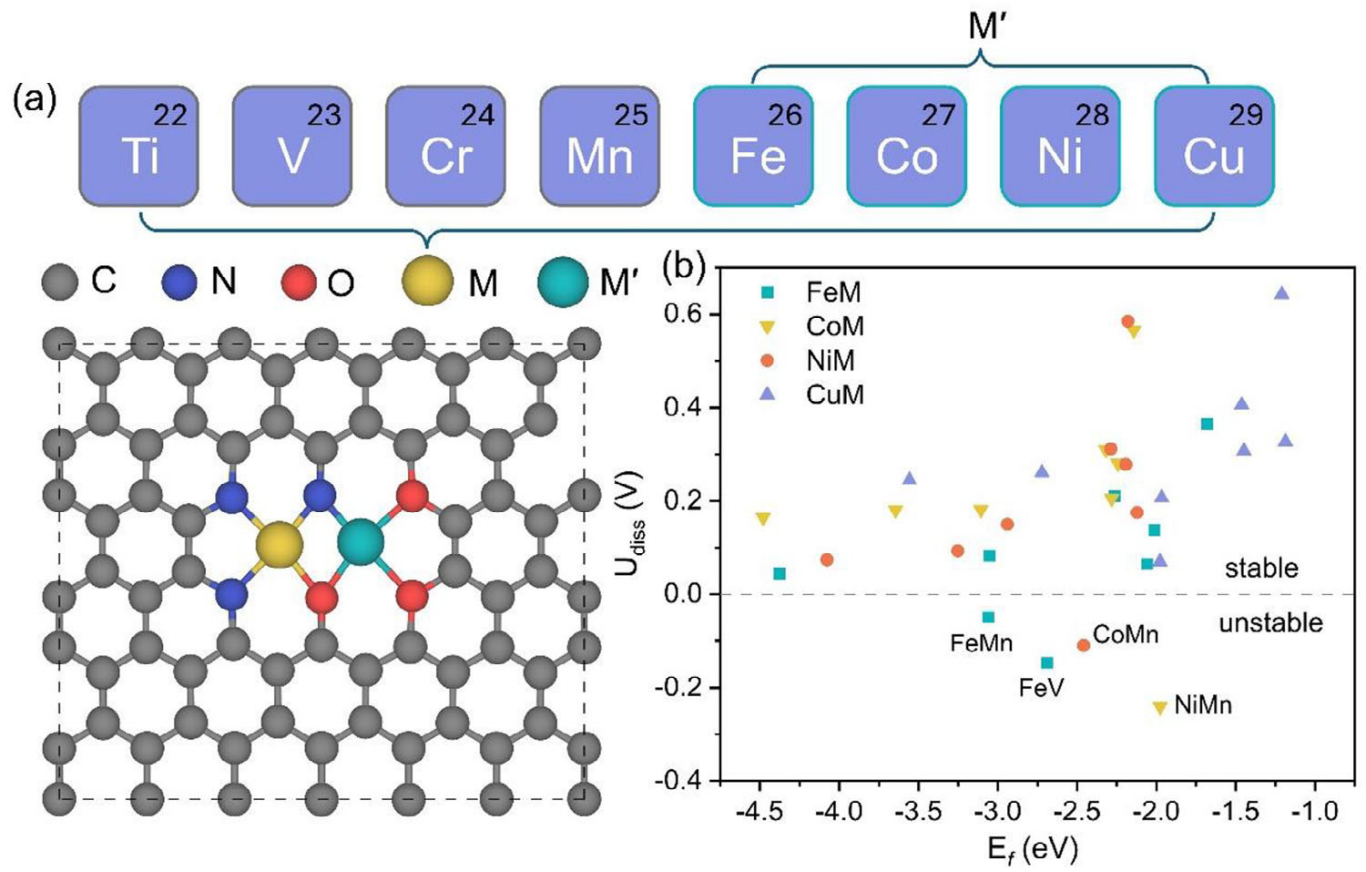
a) Structural prototype of M′M@N_3_O_3_@C (J‐M'M) nanosheet. b) Computed formation energy (E*_f_*) and dissolution potential (U_diss_) of metal dimers on N,O‐doped graphene.

32 Janus DACs (simplified J‐M'M in the following) are designed for electrochemical CO_2_R, in which M’ (Fe, Co, Ni, Cu) are coordinated with N and M (Ti, V, Cr, Mn, Fe, Co, Ni, Cu) are coordinated with O (**Figure** **1**a). Then, their thermodynamic, electrochemical, and thermal stabilities are examined by the formation energy (E*_f_*), dissolution potential (U_diss_), and AIMD simulation, respectively (Figure 1b; Figure S1 and Table S1–S4, Supporting Information). According to the definition, the negative E*_f_* and positive U_diss_ values indicate that the catalyst is thermodynamically and electrochemically stable. Obviously, the computed E*_f_* values of all Janus DACs are well below zero, suggesting their high thermodynamic stability. Furthermore, the calculated U_diss_ values rule out four systems with the positive values (J‐FeMn, J‐FeV, J‐CoMn, J‐NiMn), which are electrochemically unstable. Next, we select the J‐FeTi (with the biggest E*_f_*) and J‐CuCo (with the smallest U_diss_) to check their thermal stability under ambient conditions with the AIMD simulations (Figure S1, Supporting Information). The total energy displays periodic oscillations near the equilibrium state, and the structure exhibits no significant deformation during the entire simulation period of 10 picosecond (ps) at 300 K, demonstrating their thermal stability. To further explore the stability of coordinated oxygen, J‐FeCo was selected as a model system to investigate the possibility for the coordinated oxygen to be further hydrogenated and reduced to H_2_O. The AIMD simulation for more than 12‐ps was first conducted in a solvent environment to equilibrate the system (Figure S2a, Supporting Information). Subsequently, the kinetic barriers for the adsorption of H to coordinated oxygen, sourced from both free and adsorbed H_2_O, were evaluated (Figure S2b–f, Supporting Information). The kinetic barrier for the first hydrogenation step of the three coordinated oxygen species is sufficiently high, suggesting that the coordinated oxygen is highly difficult to be removed under electrochemical reduction conditions. During the electrochemical CO_2_R process, the active site may be blocked by the strong adsorption of *OH and *H_2_O in an aqueous solution.^[^ ^40^ ^]^ The energy barrier criterion of 0.75 eV is commonly used to define a fast electrochemical process, and the reaction with energy barrier larger than 0.75 eV is kinetically unfavorable.^[^ ^41^ ^]^ The adsorption energy larger than 0.75 eV here is considered difficult to remove the *OH and *H_2_O species from the surface, thus poisoning the active sites. Therefore, the adsorption energies of *OH and *H_2_O on both two sites for the rest of Janus DACs are calculated, and the stronger adsorption energies of *OH and *H_2_O at one of the sites were displayed (Figure S3, Supporting Information). J‐FeTi, J‐FeCu, J‐CoTi, J‐CoV, J‐NiTi, J‐NiV, J‐CuTi, J‐CuV, and J‐CuNi are discarded for the next step, as the removal of *OH is difficult due to the high adsorption energy. Additionally, the co‐adsorption of reaction species on the opposite side is not considered, as the graphene‐based catalysts synthesized in experiments are usually multi‐layered.^[^ ^40^ ^]^ Notably, we carefully confirmed the structures with nonmagnetic (NM), ferromagnetic (FM), and antiferromagnetic (AFM) configurations to obtain the ground state (Table S5, Supporting Information). The ground states for most Janus DACs (J‐FeCo, J‐FeFe, J‐CoCr, J‐CoFe, J‐CoCo, J‐CoCu, J‐NiCr, J‐NiFe, J‐NiNi, J‐CuMn, J‐CuFe, J‐CuCo, J‐CuCu) are FM. The optimized structures show that metal atoms and an oxygen atom slightly protrude from the planar structure (Figure S4, Supporting Information).

### 2.2 CO_2_R vs HER and COOH vs OCHO

**Figure 2:**
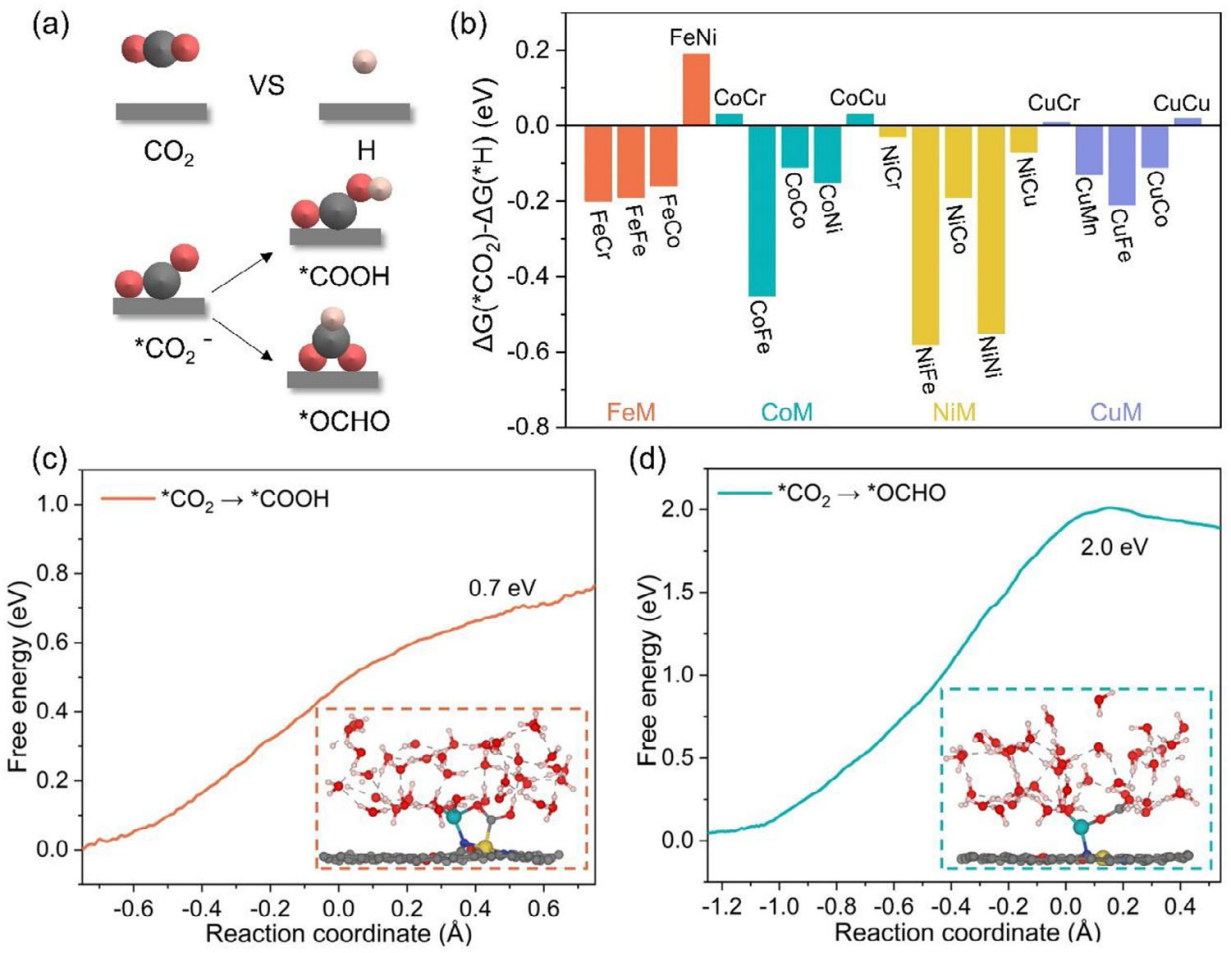
a) Schematic illustration for the selectivity of CO_2_ or H adsorption, CO_2_R to *COOH or *OCHO. b) The difference in adsorption free energy between *CO_2_ and *H. The kinetic barrier of CO_2_R to c) *COOH and d) *OCHO. The insets represent snapshots of the transition states.

The adsorption of inert CO_2_ is the first and critical step in the CO_2_R process. The rest 19 Janus DACs can be divided into two categories depending on the adsorption configuration of CO_2_ (Figure S5, Supporting Information). CO_2_ chemisorption occurs on J‐FeFe, J‐CoFe, and J‐NiFe with C and O atoms interacting with Fe site. The rest of the Janus DACs interact with CO_2_ by physisorption, where the adsorption energy (E_ads_) values are close to 0 eV (Table S6, Supporting Information). Since the electrochemical reduction of CO_2_ is carried out in aqueous solution, the hydrogen evolution reaction (HER) is the main competitive reaction and needs to be suppressed. Here, the competition between CO_2_R and HER could be evaluated with more stable CO_2_ adsorption (**Figure** **2**a,b). If the adsorption of CO_2_ is stronger than that of *H, the CO_2_R shall dominate. 14 Janus DACs (J‐FeCr, J‐FeFe, J‐FeCo, J‐CoFe, J‐CoCo, J‐CoNi, J‐NiCr, J‐NiFe, J‐NiCo, J‐NiNi, J‐NiCu, J‐CuMn, J‐CuFe, and J‐CuCo) are screened out because they favor CO_2_ adsorption.

The initial hydrogenation of CO_2_ leads to the formation of either *COOH or *OCHO, which usually influences the selectivity toward CO or HCOOH.^[^ ^42^, ^43^ ^]^ From a thermodynamic viewpoint, the formation of *OCHO seems more favorable than *COOH on some systems (Figure S6, Supporting Information). Additionally, taking J‐FeCo as an example, the adsorption free energy of *OCHO is lower than that of *COOH under CO_2_R working potentials in both acidic and alkaline conditions (Figure S7, Supporting Information).

However, CO was identified as the main product on almost all SACs, homonuclear and heteronuclear DACs experimentally, although some catalysts endow a thermodynamic preference for HCOO* in theoretical calculations.^[^ ^44^ ^]^ This indicated that a direct comparison of *COOH and HCOO* binding strengths is not enough to decide the selectivity of *COOH or *OCHO due to the missing kinetic information. Li et al. proposed that oxygen doping on Sn‐SAC breaks the uniform charge distribution and promotes V‐shaped CO_2_ chemisorption, facilitating the formation of kinetically dominant *COOH intermediate.^[^ ^45^ ^]^ Therefore, we employed the slow‐growth method with explicit water model to sample the energy change during the hydrogenation of *CO_2_ to *COOH or *OCHO by considering the microenvironment in electrocatalysis on J‐FeCo (Figure 2c,d). Firstly, 10‐ps AIMD is performed to equilibrate the systems with the adsorption of CO_2_ in explicit water (Figure S8, Supporting Information). Obviously, the total energy for V‐shaped CO_2_ chemisorption is lower than that for physisorption, which is a metaphor for the favorable configuration to *COOH.^[^ ^44^, ^46^ ^]^ Moreover, the hydrogenation of CO_2_ to COOH is kinetically favorable with an energy barrier of 0.7 eV, while a larger energy barrier (2 eV) needs to be overcome for *OCHO (Figure 2c,d). Therefore, the pathway of *OCHO is not considered in the following discussion. By the way, it can be seen that Co atom protrudes from the catalyst surface, connecting to both the adsorbate and coordinated nitrogen. This configuration likely stems from multiple factors, such as intermediate adsorption and the spontaneous extraction of protons from the electrolyte required for OH to reform into H₂O, ultimately pulling the Co atom upward the adsorption of H₂O on Co. Nevertheless, AIMD simulations conducted over 20‐ps in a neutral environment demonstrate that both systems exhibit considerable stability. The stability of Co─N bond will be discussed in the following. Additionally, the nearly invariant average ICOHP values (−1.90, −1.91, and −1.80 eV) for the Co─N bonds of pristine surface (Figure S2a, Supporting Information), OCHO‐adsorbed surface (Figure S9a, Supporting Information), and COOH‐adsorbed surface (Figure S9b, Supporting Information) further confirm the robust stability of the Co─N bond under reaction conditions.

### 2.3 Catalytic Activity and Product Distribution of CO_2_R

**Figure 3:**
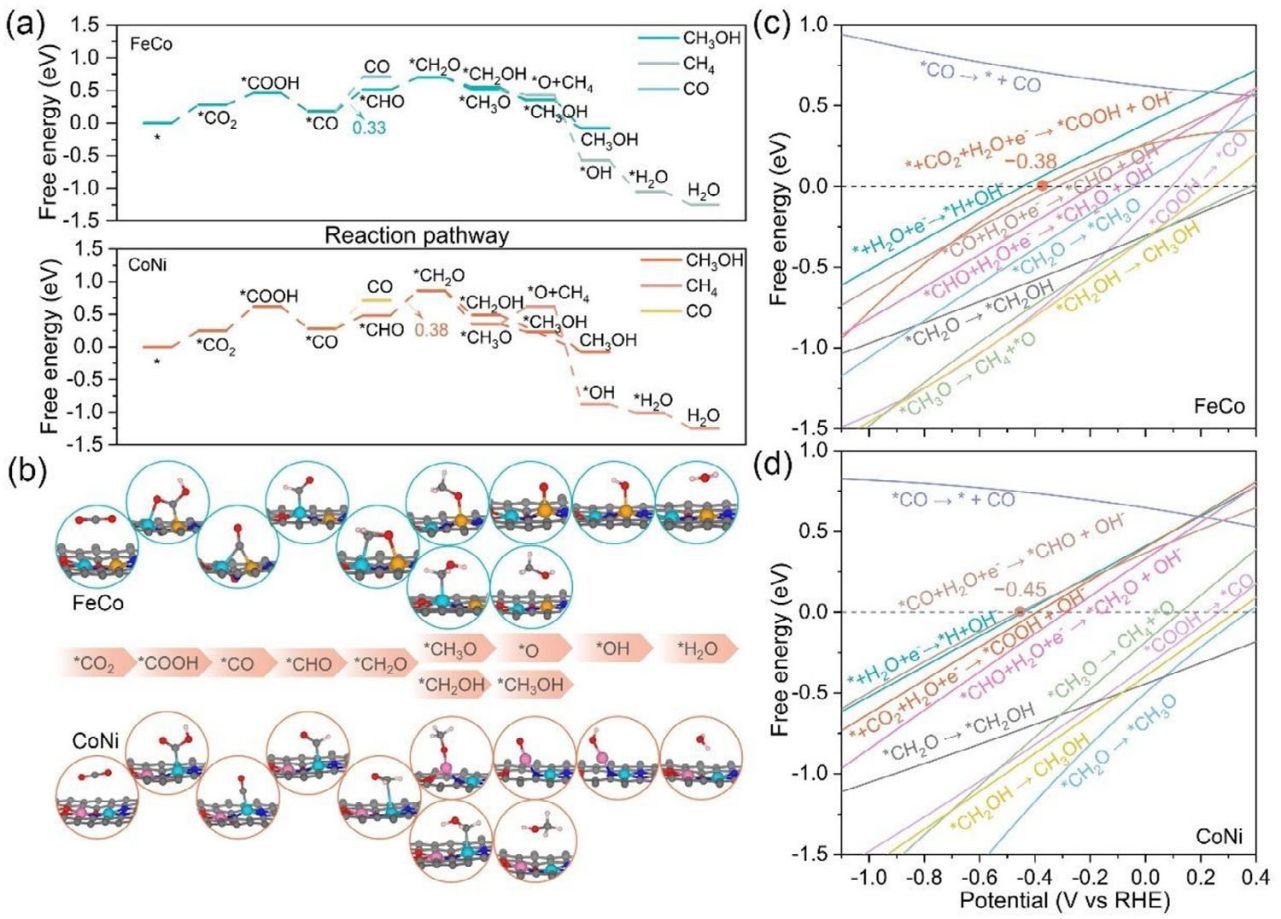
a) Free energy diagrams of CO_2_ reduction to CH_4_ and CH_3_OH on J‐FeCo and J‐CoNi. b) Optimized adsorption configurations of intermediates during the CO_2_R on J‐FeCo and J‐CoNi. Free energy changes of elementary reactions of the CO_2_R and HER as a function of potential on c) J‐FeCo and d) J‐CoNi. The yellow and green numbers represent the limiting potential to produce methanol and methane, respectively.

Based on the aforementioned discussion, we further investigated the CO_2_R pathway and examined the catalytic activities for the rest 14 Janus DACs. The CO_2_R process is convoluted with multiple proton‐coupled electron transfer steps. All possible reaction routes were considered on these systems, and we just presented the energetically preferred route for specific products (Figure S6 and S10, Supporting Information). In the next steps, the hydrogenation on *COOH always leads to the formation of *CO by releasing one H_2_O molecule. It is well known that *CO is a crucial intermediate in the CO_2_R process to form other C1 products except HCOOH, the adsorption strength of which is closely related to product selectivity.^[^ ^40^ ^]^ The weak *CO adsorption leads to direct desorption of CO under low electrode potentials. Conversely, a stably adsorbed *CO should be capable of further reduction. Remarkably, the formation of *CHO from *CO is more favorable energetically than *CO desorption for all considered Janus DACs, rendering C1 hydrocarbons rather than CO (**Figure** **3**a; Figure S61, Supporting Information). Theoretically, the intrinsic activity of electrocatalyst can be estimated by the limiting potential (U_L_). The U_L_ values and products of CO_2_R for 14 Janus DACs are summarized (**Table** **1**). Consistent with the rate‐determining step (RDS) for the production of C1 hydrocarbons over most catalysts,^[^ ^47^, ^48^, ^49^ ^]^ *CO + H^+^ + e → *CHO is the RDS of CO_2_R to CH_3_OH and CH_4_ for most of Janus DACs (Table 1). Then *CHO is further hydrogenated to generate *CH_2_O, which is more prone to be hydrogenated to *CH_3_O than *CH_2_OH. The *CH_3_O intermediate is capable of undergoing hydrogenation to form CH_3_OH or hydrogenation and dehydration to yield CH_4_. According to the Arrhenius equation, *k* = *Ae* ^−ΔG/*RT*^, the production of CH_3_OH is favored if ΔG (*CH_3_O → *CH_3_OH) − ΔG(*CH_3_O → *CH_4_) > 0.10 eV. To summarize, the moderate *CO binding strength enables almost all the 14 Janus DACs to produce CH_3_OH or CH_4_, where J‐FeCo and J‐CoNi exhibit the highest activity for the CH_3_OH and CH_4_ because of the lowest U_L_ of −0.33 and −0.38 V, respectively (Figure 3a,b).

**Table 1:** The rate‐determining step, theoretical limiting potential, and corresponding products of CO_2_R for the Janus DACs.

J‐M'M	Rate‐determining step	U_L_ (V)	Products
FeCr	*CO → *CHO	−0.56	CH_4_
FeFe	*OH → *H_2_O	−0.68	CH_4_
FeCo	*CO → *CHO	−0.33	CH_4_ CH_3_OH
CoFe	*CO → *CHO	−0.61	CH_4_
CoCo	*CO → *CHO	−0.69	CH_4_
CoNi	*CHO → *CH_2_O	−0.38	CH_3_OH
NiCr	*CO_2_ → *COOH	−0.26	CO
NiFe	*CO → *CHO	−0.93	CH_4_
NiCo	*CO → *CHO	−0.73	CH_4_
NiNi	*CO → *CHO	−0.73	CH_3_OH
NiCu	*CO → *CHO	−1.26	CH_3_OH
CuMn	*CH_3_O → *CH_3_OH	−0.59	CH_3_OH
CuFe	*CO → *CHO	−0.95	CH_4_
CuCo	*CO → *CHO	−0.69	CH_4_

The catalyst actually works under the applied electrode potential, which can affect chemical reactivity.^[^ ^50^ ^]^ Moreover, the pH and solvent effects are of great importance.^[^ ^50^ ^]^ To precisely capture the adsorption energy of intermediates, electrocatalytic simulations conducted in a more realistic environment—accounting for both solvent effect and the applied potential—are crucial for gaining a reliable atomic‐level insight into reaction mechanism. Therefore, we have re‐simulated the CO_2_R process by considering the electrode potential and the implicit solvent model in constant‐potential free energy calculations (Figure 3c,d; Figure S11, Supporting Information). The main products for CO_2_R are still CH_3_OH and CH_4_ on J‐FeCo and J‐CoNi. In addition, the adsorption of *CO is enhanced with the applied negative potential, which hinders the desorption of CO and facilitates its hydrogenation. It is worth noting that the RDS of the reaction path is changed from *CO + H^+^ + e^−^ → *CHO to * + CO_2_ + H^+^ + e^−^ → *COOH on J‐FeCo with a limiting potential of −0.38 V vs. RHE. And the RDS changes from the elementary step of *CHO + H^+^ + e^−^ → *CH_2_O to *CO + H^+^ + e^−^ → *CHO with a limiting potential of −0.45 V vs. RHE for J‐CoNi. As the limiting potential after considering charge and solution effects is still low, the production of CH_3_OH and CH_4_ under room temperature is favorable.^[^ ^51^, ^52^ ^]^ These interesting results lead to two questions: 1) why can DACs with oxygen coordination produce CH_3_OH and CH_4_ since the final product for most SACs and DACs in CO_2_R is CO? and 2) whether and how does oxygen coordination enhance the adsorption of CO?

### 2.4 Comparison between Normal DACs and Janus DACs

**Figure 4:**
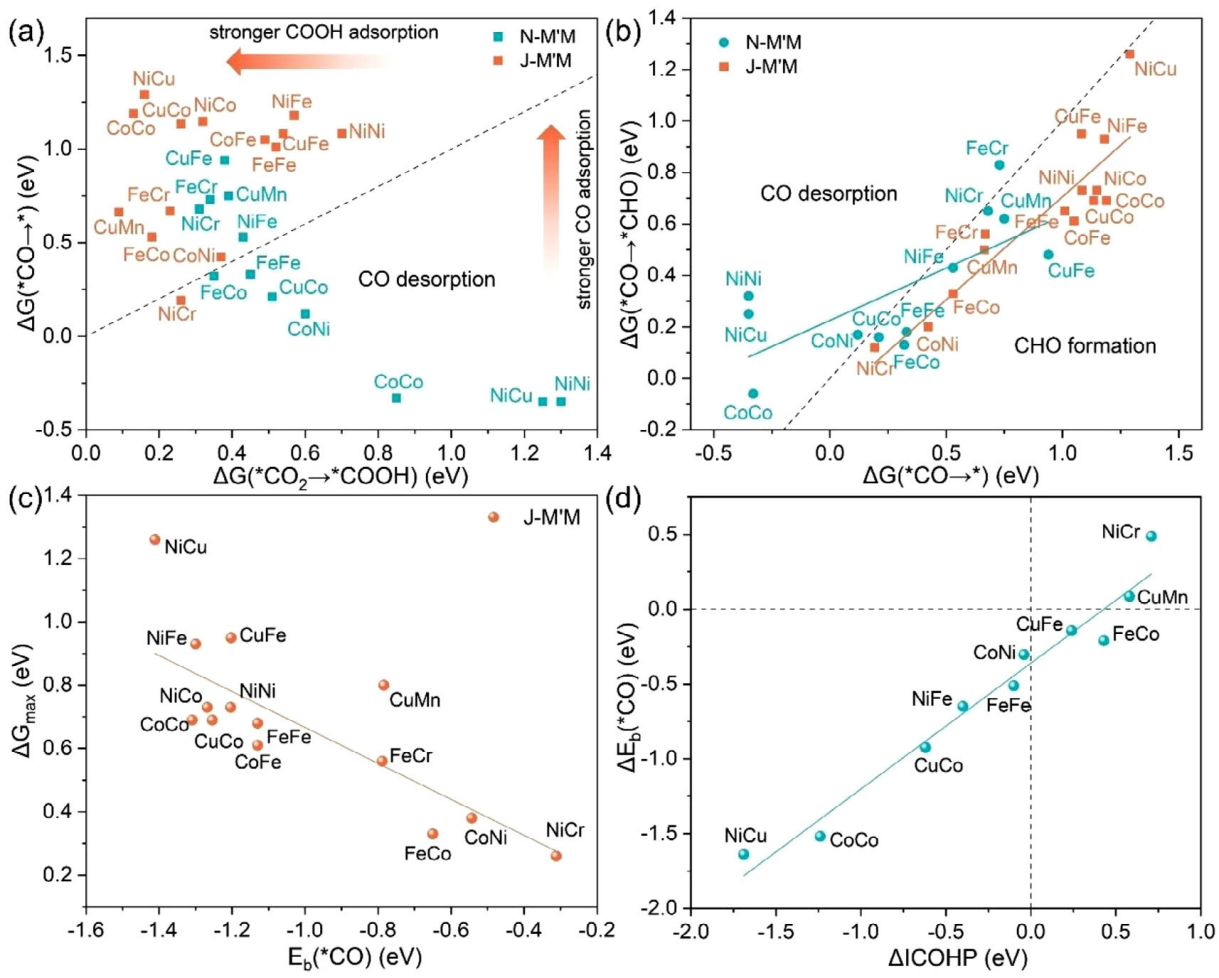
a) The free energy change of CO_2_ hydrogenation to *COOH and *CO desorption, b) *CO desorption and *CO hydrogenation to *CHO on Janus DACs and normal DACs. c) Relationship between ΔG_max_ and E_b_(*CO) on Janus DACs. d) Relationship between ΔICOHP = ICOHP(J‐M'M) − ICOHP(N‐M'M) and ΔE_b_(*CO) = E_b_(J‐M'M*CO) − E_b_(N‐M'M*CO).

For the first question, 13 identical combinations of DACs with only nitrogen coordination (M'M‐N_6_, simplified N‐M'M) are investigated for comparison. Generally, one of the two elementary steps, either *CO_2_ → *COOH or *CO → *CHO, in the pathways for the C1 production is typically the RDS and governs the overall catalytic efficiency of CO_2_R. Therefore, we first compare these two critical elementary reactions between normal DAC and Janus DAC, where the active site of normal DACs is the same as those of Janus DAC. ΔG(*CO_2_ → *COOH) describes the ability to reduce CO_2_, while ΔG(*CO → *CHO) reflects the capability for CO hydrogenation. The plot of ΔG(*CO_2_ → *COOH) versus ΔG(*CO → *) shows that normal DACs have larger ΔG(*CO_2_ → *COOH) than ΔG(*CO → *), indicating that the *CO molecules tend to desorb from the surface (**Figure** **4**a). In contrast, Janus DACs, except the J‐NiCr, shows that the ΔG(*CO_2_ → *COOH) is smaller than ΔG(*CO → *), leading to strong *CO adsorption. Additionally, Janus DACs exhibit stronger adsorption of COOH and CO compared to normal DACs. Then, we investigated the *CO + H^+^ + e^−^ → *CHO step (Figure 4b). By comparing ΔG(*CO → *CHO) with ΔG(*CO → *), we find that the *CO molecules on all Janus DACs are more likely to undergo further hydrogenation rather than desorption, whereas *CO on many normal DACs is more prone to desorb thermodynamically. Overall, Janus DACs endow two advantages over normal DACs: 1) favorable CO_2_ hydrogenation and 2) high CO adsorption strength that promotes further CO hydrogenation.

The binding energies of *COOH, *CO, and *CHO intermediates are closely related to these two elementary reactions mentioned above. It is generally recognized that a linear scaling relationship between the adsorption energies of intermediates exist in most catalysts, which hinders the simultaneous optimization of the adsorption strength of different intermediates during the CO_2_R process and leads to high overpotentials.^[^ ^17^, ^53^, ^54^ ^]^ Remarkably, there is a linear scaling relationship among the adsorption energies of *COOH, *CO, and *CHO on normal DACs, while this relationship is clearly broken on Janus DACs (Figure S12, Supporting Information). This suggests that the binding energies of *COOH, *CO, and *CHO are no longer correlated on Janus DACs because of the oxygen coordination. Although the adsorption configurations of these intermediates on normal DACs and Janus DACs are similar, the different electronic properties caused by different coordination environments may result in significant differences in the adsorption energies of the intermediates. In the CO_2_R process, identifying appropriate descriptors has become a widely adopted approach for elucidating the mechanism. The CO adsorption energy is often used as an effective descriptor for evaluating the CO_2_R performance.^[^ ^28^, ^55^ ^]^ As expected, the catalytic activity of Janus DACs is closely related to the CO binding energy (Figure 4c). The bonding strength between the C in *CO and the active sites on DACs can be assessed through the crystal orbital Hamilton population (COHP) analysis, where the integrated COHP (ICOHP) values provide a direct evaluation of the interaction strength. Both normal DACs and Janus DACs have a linear correlation between the CO adsorption energy and ICOHP, where most Janus DACs show stronger CO bonding strength (Figure S13, Supporting Information). Correspondingly, the difference in ICOHP values between Janus DACs and normal DACs is proportional to the difference in their CO adsorption energies (Figure 4d). Interestingly, the covalent interaction between CO and the active sites on J‐FeCo, and J‐CuFe is relatively weak, but their CO adsorption energies remain stronger than those on normal DACs. One of the reasons is that CO occupies the bridge sites on J‐FeCo while the top site of the Fe on N‐FeCo. The values ​​taken by ICOHP are both the bonding between the Fe site and C of CO. Since J‐FeCo and J‐CoNi show the best CO_2_R performance, we next address the mechanism for enhanced CO adsorption strength on oxygen‐coordinated DACs.

### 2.5 Intrinsic Catalytic Origin

**Figure 5:**
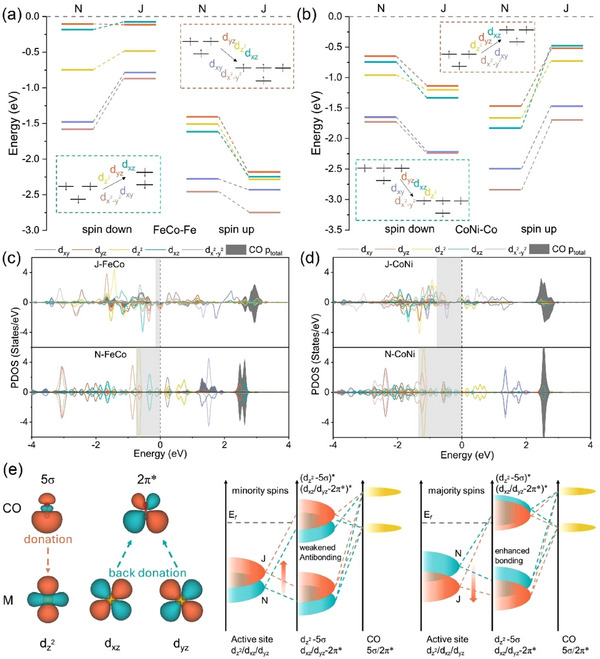
The energy level diagrams for 3d orbitals referring to the center of each d band: a) Fe of J‐FeCo and N‐FeCo, and b) Co of J‐CoNi and N‐CoNi. The abbreviations N and J at the top represent normal DAC and Janus DAC respectively. The horizontal dashed lines represent the Fermi level. The insets qualitatively depict the energy levels and electron rearrangement. Partial density of states (PDOSs) of the c) Fe, Co 3d, d) Co, Ni 3d orbitals and the adsorbed *CO p orbital. e) Schematic illustration of orbital interactions between the active site, M‐3d_yz_/d_z2_/d_xz_ orbitals, and 5σ and 2π* of adsorbed *CO.

The d‐band center and charge transfer have been widely recognized as effective descriptors for predicting catalyst activity.^[^ ^56^, ^57^ ^]^ However, these approaches fail to describe SACs and single atom alloys (SAAs), where the active sites are isolated to each other.^[^ ^58^, ^59^ ^]^ In the case of these Janus DACs, neither the d‐band center nor charge transfer can effectively predict catalytic activity (Figure S14, Supporting Information). It has been reported that frontier molecular orbitals and narrow d‐band levels play a critical role in determining the behavior of adsorbates on SACs or SAAs.^[^ ^60^, ^61^ ^]^ Consequently, we consider the d‐band energy levels of the metal sites and the Partial Density of States (PDOSs) for depicting the interaction between CO and the active sites to reveal the mechanism. According to the crystal field model, the d‐orbital energy level of transition metal is related to their coordination environment. Compared to normal DACs, the two metal sites in Janus DAC exhibit stronger spin polarization and the rearrangement of 3d orbital energy levels and electrons (Figure S15, Supporting Information). Specifically, for J‐FeCo, the spin‐down energy levels of both Fe and Co upshift toward the Fermi level compared to N‐FeCo, while the spin‐up energy levels show the opposite trend (**Figure** **5**a,b; Figure S16, Supporting Information). For J‐CoNi, the spin‐up energy levels of Co and Ni move toward the Fermi level relative to N‐FeCo, whereas the spin‐down levels display opposite way (Figure 5a,b; Figure S16, Supporting Information). This evidence corroborates that the oxygen coordination optimizes the filling of the 3d orbitals of the two metals.^[^ ^27^ ^]^ Additionally, the spin‐split orbitals can match with intermediates well, favoring stronger adsorption. The PDOSs of d orbitals for metal sites and p orbitals for adsorbed CO show that the orbital overlap between CO and metal sites on normal DACs is smaller than Janus DACs, resulting in weaker adsorption (Figure 5c,d). It is reported that the bonding between CO and transition metals is a two‐step process: (1) 5σ→d_z2_ donation, which contributes to the M‐C bond and enhances the *CO binding strength and (2) d_xz_, d_yz_→2π* back‐donation, which weakens the C≡O bond and thereby activates the *CO (Figure 5e; Figure S17, Supporting Information).^[^ ^62^ ^]^ It is clear that the d_xy_ and d_x2‐y2_ orbitals poorly match with the 5σ and 2π* of CO, thus contributing negligibly to the bonding between the active site and carbon (Figure 5c,d). Due to the high electron density, the bonding orbitals are usually dominated by the majority spins. The majority‐spin orbital energy levels of Fe in J‐FeCo and Co in J‐CoNi shift downward compared to those in N‐FeCo and N‐CoNi, respectively (Figure 5a,b). This adjustment facilitates effective overlap with the orbitals of adsorbed molecules, fostering the formation of stable chemical bonds (Figure 5c–e; Figure S18, Supporting Information). The antibonding orbitals are primarily contributed by minority spins, with the portion below the Fermi level weakening the adsorption strength. The minority‐spin orbital energy levels of Fe in J‐FeCo and Co in J‐CoNi shift upward relative to those in N‐FeCo and J‐CoNi (Figure 5a,b). This change correspondingly reduces the contribution of antibonding orbitals below the Fermi level and further promotes the adsorption process (Figure 5c–e; Figure S18, Supporting Information). It is worth noting that the binding strength between oxygen‐coordinated transition metals and oxygen is lower than that between metals and nitrogen (Figure S19, Supporting Information). These changes in the catalyst's crystal field typically affect the spin state of the transition metal active sites.^[^ ^57^ ^]^ Accordingly, their CO adsorption behavior may also be related to the spin state of the metal. We find that metals in oxygen‐coordinated DACs experience the transition from low‐spin (LS) to high‐spin (HS) states (Table S7, Supporting Information). The larger magnetic moment of high‐spin metals promotes orbital overlaps between intermediates and metal active sites, facilitating the formation of spin channels for electron transfer and thereby boosting adsorption. Besides, the Fe in J‐FeCo, being in a high‐spin (HS) state, experiences a reduction in spin‐down electrons, which implies fewer electrons are available to form antibonding orbitals (Figure 5a,c).

By the way, the relatively weak binding strength between metal M and coordinated oxygen may lead to the breaking of M‐O bonds under working conditions, but this metal can still strongly bind to a nitrogen atom (Figure S19c, Supporting Information). The stability of the catalyst is evaluated by the energy barriers for breaking the Co─N bond under solvent conditions and across varying CO coverages (Figure S20, Supporting Information). The substantial kinetic barrier required to break the Co─N bond demonstrates the remarkable stability of the catalyst structure. In addition, the dangling metal sites have also been demonstrated to remain dynamically stable and promote CO_2_R process.^[^ ^63^ ^]^ Although increased CO coverage may weaken the Co‐N bond, our findings indicate that the kinetic barrier for Co‐N bond cleavage remains substantially high even at high CO coverage levels. Further experimental and theoretical studies are necessary to systematically investigate specific adsorbate coverage levels under varying working potentials and their impact on CO_2_R performance.

### 2.6 An Activity Descriptor for CO_2_ Reduction on Janus DACs

As discussed above, a notable linear correlation exists between the CO adsorption energy and the ΔG_max_ of Janus DACs. Based on intrinsic atomic properties, a descriptor can be identified to predict CO adsorption energy and establish structure‐activity relationships, so that we can roughly estimate the impact of other potential combinations on CO_2_R performance. The effective descriptor would significantly streamline the screening of desirable catalysts, eliminating the need for intricate computations. This is particularly valuable since experimentally measuring CO adsorption energy poses considerable challenges. Inspired by prior studies,^[^ ^64^, ^65^ ^]^ we selected two intrinsic atomic properties (d‐electron numbers and electronegativity) as key determinants, with CO adsorption energy as the target property. The d‐electron number of the metal active sites is always listed as one of the most important features. Furthermore, to capture the interactions between different metals and their surrounding coordination environments, we incorporated the electronegativity of both the metal and its coordinating atoms, as electronegativity quantifies the electron affinity of distinct atoms. Following the approach reported by Ren et al.,^[^ ^66^ ^]^ we adopted the ratio χ_M’/M_/χ_O/N_ to characterize the interaction between the metal atom and its coordinating neighbors, which represents the relative electronegativity.

**Figure 6:**
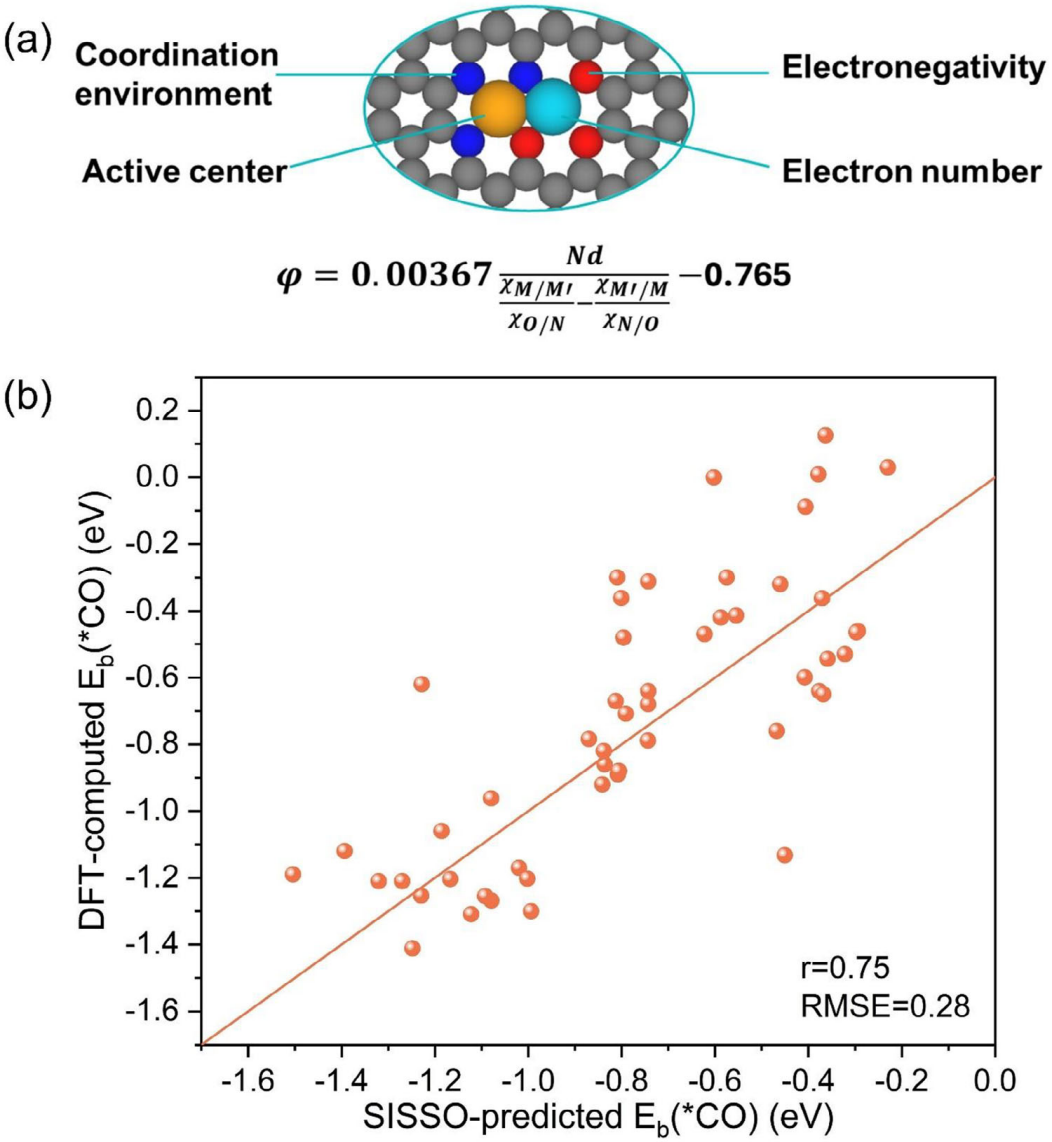
a) Schematic of the proposed descriptor which is described by the intrinsic atomic properties with electronegativity (χ) and the number of d electrons (N_d_). The first term of the denominator represents the electronegativity of the metal adsorbed intermediates and the coordinating atom on one side, and the second term represents the electronegativity of the metal and coordinating atom on the other side. b) Comparison of DFT‐computed and SISSO‐predicted E_b_(*CO) for Janus DAC considering both active sites.

To simplify the descriptor, we employed the SISSO model,^[^ ^67^ ^]^ which has the advantage of conducting exhaustive search within the solution space using a small training dataset to provide a low‐complexity descriptor. Based on the aforementioned 28 stable systems, each containing two active sites for a total of 56 datasets, we derived a simple yet effective descriptor, φ = $\;0.00367\frac{N_{d}}{\frac{\chi_{M^{\prime }/M}}{\chi_{O/N}}-\frac{\chi_{M/M^{\prime }}}{\chi_{N/O}}}-0.765$ (**Figure** **6**a). The descriptor φ exhibits a relatively high Pearson correlation coefficient (r) and a small root‐mean‐square error (RMSE), signifying its ability to predict CO adsorption energy (Figure **6**b). Therefore, this descriptor, similar to the one mentioned by Ren et al.,^[^ ^66^ ^]^ can also be referred to as the “effective d electron number”, which physical meaning can be interpreted as the redistribution of d‐electrons of the metal active site as influenced by the surrounding coordination environment. It can be observed that different metal combinations result in a wide range of variations in the binding strength between the metal active sites and CO (Figure S13, Supporting Information). The value of φ depends on the d‐electron number and the difference in relative electronegativity between the two metals. For metal active sites with the same d‐electron number, even a minor difference of ≈0.07 in the relative electronegativity between the two sides can lead to an E_b_(*CO) difference of ≈0.6 eV, as seen in the cases of CoFe‐Co and CoCo‐Co2 (Table S8, Supporting Information). Conversely, when the relative electronegativity difference is similar, the E_b_(*CO) values also remain close, such as CoFe‐Co and CoNi‐Co1 (Table S8, Supporting Information). Moreover, the number of d‐electrons, as the numerator in the φ expression, indeed exerts a significant influence on the adsorption energy when it varies. However, the Pearson correlation coefficient of this descriptor is not very high, leading to insufficient transferability to other systems.

## 3 Conclusion 

To summarize, we designed 32 Janus dual‐atom catalysts (DACs) with N_3_O_3_ hybrid coordination and assessed their CO_2_ reduction (CO_2_R) performance using DFT calculations. Thirteen Janus DACs selectively produce CH_3_OH or CH_4_, in contrast to conventional N‐coordinated DACs, which primarily yield CO. J‐FeCo and J‐CoNi exhibit exceptional activity with favorable limiting potentials of −0.38 and −0.45 V vs. RHE, respectively. Enhanced *COOH and *CO adsorption in Janus DACs promotes CO_2_ and CO hydrogenation compared to normal DACs. Mechanistic studies reveal that the upshift/downshift of minority‐/majority‐spin energy levels of d_xz_, d_z2_, and d_yz_ orbitals toward the Fermi level, driven by oxygen coordination, and the transition from low‐spin to high‐spin states at the metal site, enhance CO‐metal orbital interactions. We propose an effective descriptor, φ, based on the intrinsic atomic properties of the catalytic site and coordination environment, to predict *CO adsorption energy and CO_2_R performance. Our findings show that Janus DACs favor CH_3_OH and CH_4_ production over CO and establish a clear structure‐activity relationship through the proposed descriptor.

## 4 Computational Details

All spin‐polarized DFT calculations were performed using the Vienna ab initio Simulation Package (VASP) code.^[^ ^68^, ^69^, ^70^ ^]^ The ion‐electron interactions were described using the projector augmented wave (PAW) functional. The electronic exchange correlation interactions was described by the Perdew−Burke−Ernzerhof (PBE) functional within the generalized gradient approximation (GGA).^[^ ^71^ ^]^ The valence electrons were described as planewaves. The energy cutoff for the planewave basis expansion was set to 500 eV for relaxations and static calculations, and 400 eV for AIMD simulations. The convergence criteria of the electronic energy and force were set to 1.0 × 10^−5^ eV and 0.02 eV Å^−1^, respectively. The Brillouin zones were sampled with a 3 × 2 × 1 Monkhorst‐Pack mesh.^[^ ^72^ ^]^ The van der Waals interactions were described by DFT‐D3 correction.^[^ ^73^ ^]^ A vacuum space along the z‐direction was set larger than 14 Å to mitigate the effects of interactions between neighboring images. The M'M‐N_3_O_3_/N_6_@C slab was composed of a 3 × 6 supercell (12.83 × 14.82 Å^2^), which is large enough to avoid the interaction between two periodic units. The crystal orbital Hamilton population (COHP) analysis was performed using the LOBSTER package.^[^ ^74^ ^]^ According to the computational hydrogen electrode (CHE) model,^[^ ^75^ ^]^ the Gibbs free‐energy changes (Δ*G*) for elementary steps were calculated as

(1) $$\Delta G=\Delta E+\Delta ZPE-T\Delta S+\int C_{p}dT$$

where Δ*E*, Δ*ZPE*, Δ*S*, and *C_p_* denote the difference of electronic energy from the DFT calculations, the zero‐point energy (ZPE), the entropy, and the heat capacity at room temperature (T = 298.15K) between products and reactants, respectively, which were obtained from the vibrational frequency calculations through the VASPKIT code.^[^ ^76^ ^]^ The limiting potential (U_L_) was calculated from

(2) $$U_{L}=-\frac{\Delta G_{max}}{e}$$

where the ∆G_max_ is the maximum free energy change among all fundamental steps for reaction pathway. More computational details regarding slow‐growth method, constant‐potential computation and SISSO model are given in the Supporting Information.

## Conflict of Interest

The authors declare no conflict of interest.

## Supporting information



Supporting Information

## Data Availability

The data that support the findings of this study are available in the supplementary material of this article.
