# Supplementary material for: Electrochemical CO2 Reduction on Janus Dual‐Atom Catalysts: Critical Role of Oxygen Coordination and an Effective Descriptor
Source: Adv Sci (Weinh). 2025 Jul 8;12(36):e07849. doi: 10.1002/advs.202507849 (PMC12463111; doi:10.1002/advs.202507849)
Supplement: Supplementary file 1 — Supporting Information [file ADVS-12-e07849-s001.docx]

Supporting Information

**Electrochemical CO_2_ reduction on Janus Dual-Atom Catalysts: Critical Role of Oxygen Coordination and an Effective Descriptor**

*Hongling Liu^1^, Zhichao Yu^1^, Jia Zhao^3^, Weng Fai Ip^2*^, Sen Lin^3^, and Hui Pan^1,2*^*

H. Liu^1^, Z. Yu^1^, J. Zhao^3^, W.-F. Ip^2*^, S. Lin^3^, H. Pan^1,2*^

^1^ Institute of Applied Physics and Materials Engineering, University of Macau, Macao SAR 999708, P. R. China

^2^ Department of Physics and Chemistry, Faculty of Science and Technology, University of Macau, Macao SAR 999078, P. R. China

^3^State Key Laboratory of Photocatalysis on Energy and Environment, College of Chemistry, Fuzhou University, Fuzhou 350116, China

*Corresponding Authors:

Andy Ip: andyip@um.edu.mo

H. Pan: huipan@um.edu.mo (email), +853 88224427 (tel.), +853-88222454 (fax)

**Slow-growth method**

To evaluate the thermal stability and kinetic barriers, we carried ab initio molecular dynamics (AIMD) simulations and a “slow-growth” method^[1]^ with explicit solvation model, which were performed in the canonical (NVT) ensemble using Nose−Hoover thermos stats,^[2]^ keeping the temperature at 300K. A 1 × 1 × 1 Monkhorst−Pack k-point grid was used for the Brillouin zone sampling. The collective variable (CV) increment was set to 0.0005 Å, and the time step was set to 1 fs. The convergence criteria for the electronic step is set to 1×10^−4^ eV, and the POMASS of H is set to 2.

**Constant-potential computations**

To obtain the energies of the system under the electrochemical reactions, the constant-potential method was adopted.^[3,4]^ The applied voltage to the electrochemical interface was simulated by adding or removing electrons to the supercell. The aqueous environment of the electrolyte was treated with a continuum dielectric model based on the VASPsol code.^[5]^ The relative permittivity was set to 78.4 to imitate an aqueous environment and the Debye screening length was set to 3 Å to represent the compensatory charge. The potential-dependent energy of the system is formulated as^[6]^

$$\begin{aligned} E=E_{VASP}-E_{F}\Delta q\#\left( 1 \right) \end{aligned}$$

where $E_{VASP}$, $E_{F}$, and $\Delta q$ represent the DFT energy tested by VASPsol, fermi level and extra numbers of electrons. The electrode potential referenced to the standard hydrogen electrode U (V vs SHE) is calculated as

$$\begin{aligned} U\left( V vs SHE \right)= -4.44-\frac{E_{F}+E_{fermishift}}{e}\#\left( 2 \right) \end{aligned}$$

where $E_{fermishift}$ is the Fermi energy shift correction. The E−U points follow a quadratic function as

$$\begin{aligned} E\left( U \right)=-\frac{1}{2}C\left( U-U_{PZC} \right)^{2}+E_{PZC}\#\left( 3 \right) \end{aligned}$$

where $U_{PZC}$, $C$, and $E_{PZC}$ refers to the potential of zero charge (PZC), the capacitance of the corresponding system, and the energy of the system at the PZC, respectively. For each adsorption configuration, the charge variation ranging from −1e to +1e in steps of +0.25/0.5e, with a minimum of 5 points to ensure a perfect parabola. Considering the effect of pH, the electrode potential relative to the reversible hydrogen electrode (RHE) can be converted by using the following equation

U_RHE_ = U_SHE_ + 0.0592pH

The pH was set to 6.8 based on experiments (0.1 mol/L KHCO_3_). The Gibbs free energy change (ΔG) in the constant potential computation was computed as follows

$\begin{aligned} \Delta G= \Delta E+ \Delta ZPE-T\Delta S+ \int C_{p}dT+eU+\Delta G_{pH}\#\left( 4 \right) \end{aligned}$

where $\Delta E$, $\Delta ZPE$, $\Delta S$, and $C_{p}$ are the change of the electronic energy from the DFT calculations, the zero-point energy (ZPE), the entropy, and the heat capacity at room temperature (T=298.15K) between products and reactants, and U is the applied electrode potential. $\Delta G_{pH}$ is the free energy correction of H^+^ and defined as $k_{B}T\times ln10\times pH$, where k_B_ is the Boltzmann constant and pH is set as 6.8.

**SISSO model**

Sure Independence Screening and Sparsifying Operator (SISSO),^[7]^ a machine learning-based feature selection algorithm, is designed to identify the most important features in a dataset, and explores the mathematical dependence of target properties on a set of input features within the framework of compressed-sensing based dimensionality reduction. The activity index in this work $\Delta G$ can be described by the absorption energy of *CO (E_b_(*CO)), thus we choose E_b_(*CO) as the target property. To generate an effective and interpretable descriptor that can predict *CO binding energies of different Janus DACs and establish a quantitative structure−property relationship based on a small data set, we selected the intrinsic atomic features (d electron number and electronegativity) of active sites and coordination atoms as input variables, which can be obtained from the element database. To obtain a simple and physically meaningful descriptor, the mathematical operator set was given as

$$\begin{aligned} Ĥ\left( m \right)= \left\{ \left( + \right), \left( - \right), \left( * \right), \left( / \right), (^{-1} \right)\}\#\left( 5 \right) \end{aligned}$$

Pearson correlation coefficients (r) and root-mean-square errors (RMSE) are used to evaluate prediction accuracy and errors for training set and test set.

**Evaluation of thermodynamic and electrochemical stabilities**

The formation energy E*_f_* is defined as^[8]^

$$\begin{aligned} E_{f} = \frac{E_{M^{'}M-N3O3@C} - E_{M^{'}} - E_{M} - E_{N3O3@C}}{2}\#\left( 6 \right) \end{aligned}$$

where E_M′M-N3O3@C_, E_M′_/E_M_, E_N3O3@C_ are the energies of Janus DACs, metal atoms in its bulk, N,O-doped substrate, respectively.

The dissolution potential (*U*_diss_) is defined as

$$\begin{aligned} U_{diss}=\frac{U_{diss^{\circ}}\left( M, bulk \right)+U_{diss^{\circ}}\left( M^{'}, bulk \right)}{2}-\frac{E_{f}}{Ne}\#\left( 7 \right) \end{aligned}$$

where $U_{diss}^{^{\circ}}$(M/M’, bulk) is the standard dissolution potential of bulk metal in aqueous solution, N is the electron number involved in the dissolution.

**Table S1.** Computed E*_f_* and U_diss_ of J-FeM. The electrochemically unstable J-FeM structures are highlighted in black font, and the rest are stable.

| **J-FeM** | **E*_f_* (eV)** | **U_diss_ (V)** |
| --- | --- | --- |
| FeTi | -4.3774 | 0.0444 |
| **FeV** | -3.0602 | -0.05 |
| FeCr | -3.053 | 0.08325 |
| **FeMn** | -2.6906 | -0.1475 |
| FeFe | -2.0593 | 0.0648 |
| FeCo | -2.0131 | 0.1383 |
| FeNi | -2.2629 | 0.2107 |
| FeCu | -1.6794 | 0.3649 |

**Table S2.** Computed E*_f_* and U_diss_ of J-CoM. The electrochemically unstable J-CoM structures are highlighted in black font, and the rest are stable.

| **J-CoM** | **E*_f_* (eV)** | **U_diss_ (V)** |
| --- | --- | --- |
| CoTi | -4.4791 | 0.1648 |
| CoV | -3.6451 | 0.1813 |
| CoCr | -3.1065 | 0.1816 |
| **CoMn** | -1.9768 | -0.2408 |
| CoFe | -2.2815 | 0.2054 |
| CoCo | -2.2423 | 0.2806 |
| CoNi | -2.3181 | 0.3095 |
| CoCu | -2.1423 | 0.5656 |

**Table S3.** Computed E*_f_* and U_diss_ of J-NiM. The electrochemically unstable J-NiM structures are highlighted in black font, and the rest are stable.

| **J-NiM** | **E*_f_* (eV)** | **U_diss_ (V)** |
| --- | --- | --- |
| NiTi | -4.0769 | 0.0742 |
| NiV | -3.2521 | 0.093 |
| NiCr | -2.9394 | 0.1499 |
| **NiMn** | -2.4582 | -0.11 |
| NiFe | -2.1217 | 0.1754 |
| NiCo | -2.1933 | 0.2783 |
| NiNi | -2.2876 | 0.3119 |
| NiCu | -2.1802 | 0.5851 |

**Table S4.** Computed E*_f_* and U_diss_ of J-CuM. All J-CuM structures are thermodynamically and electrochemically stable.

| **J-CuM** | **E*_f_* (eV)** | **U_diss_ (V)** |
| --- | --- | --- |
| CuTi | -3.5574 | 0.2444 |
| CuV | -2.7218 | 0.2604 |
| CuCr | -1.9673 | 0.2068 |
| CuMn | -1.9774 | 0.0694 |
| CuFe | -1.4489 | 0.3072 |
| CuCo | -1.1871 | 0.3268 |
| CuNi | -1.4629 | 0.4057 |
| CuCu | -1.2101 | 0.6425 |


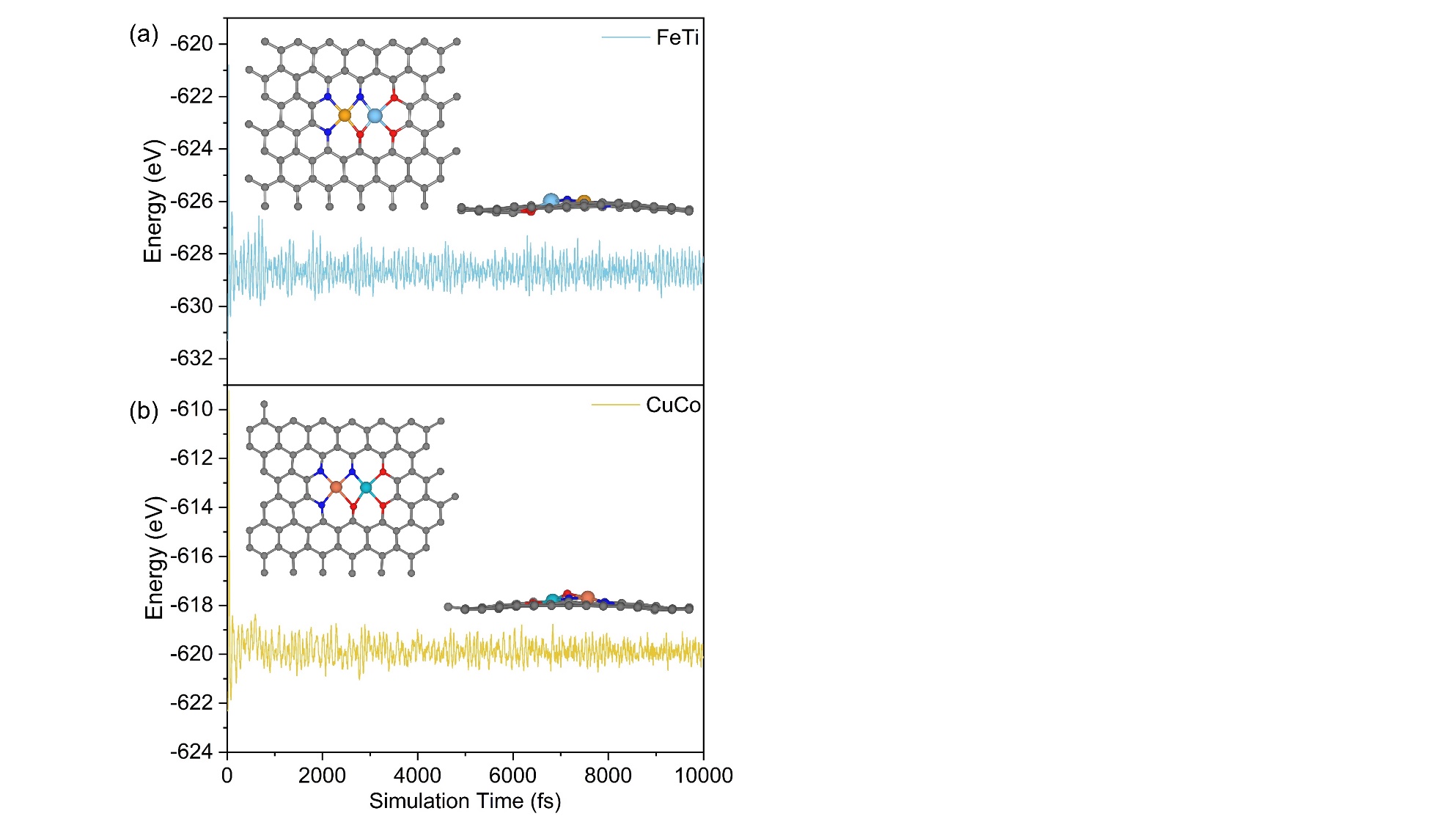


**Figure S1.** Total energy fluctuation during AIMD simulations for the J-FeTi (with the biggest E*_f_*) and J-CuCo (with the smallest U_diss_), which is performed at 300 K for 10 ps with a time step of 1 fs. The insets display the top and side views of the atomic configuration at 10 ps.

**
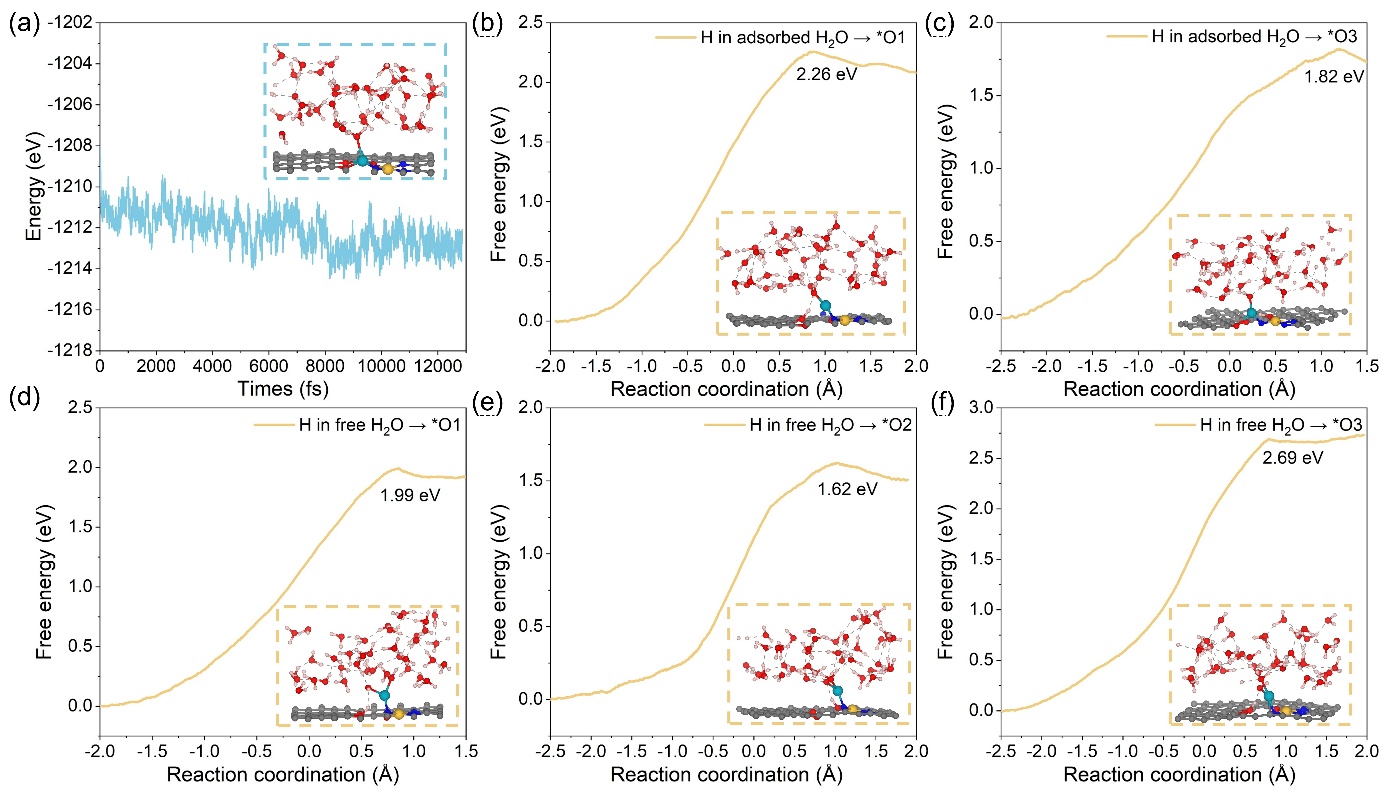
**

**Figure S2.** (a) The AIMD simulation of J-FeCo under solvent environment. The kinetic barrier for the initial hydrogenation step of the coordinated oxygen with H origined from the (b-c) adsorbed H_2_O and (d-f) free H_2_O. The insets represent snapshots of the transition states.


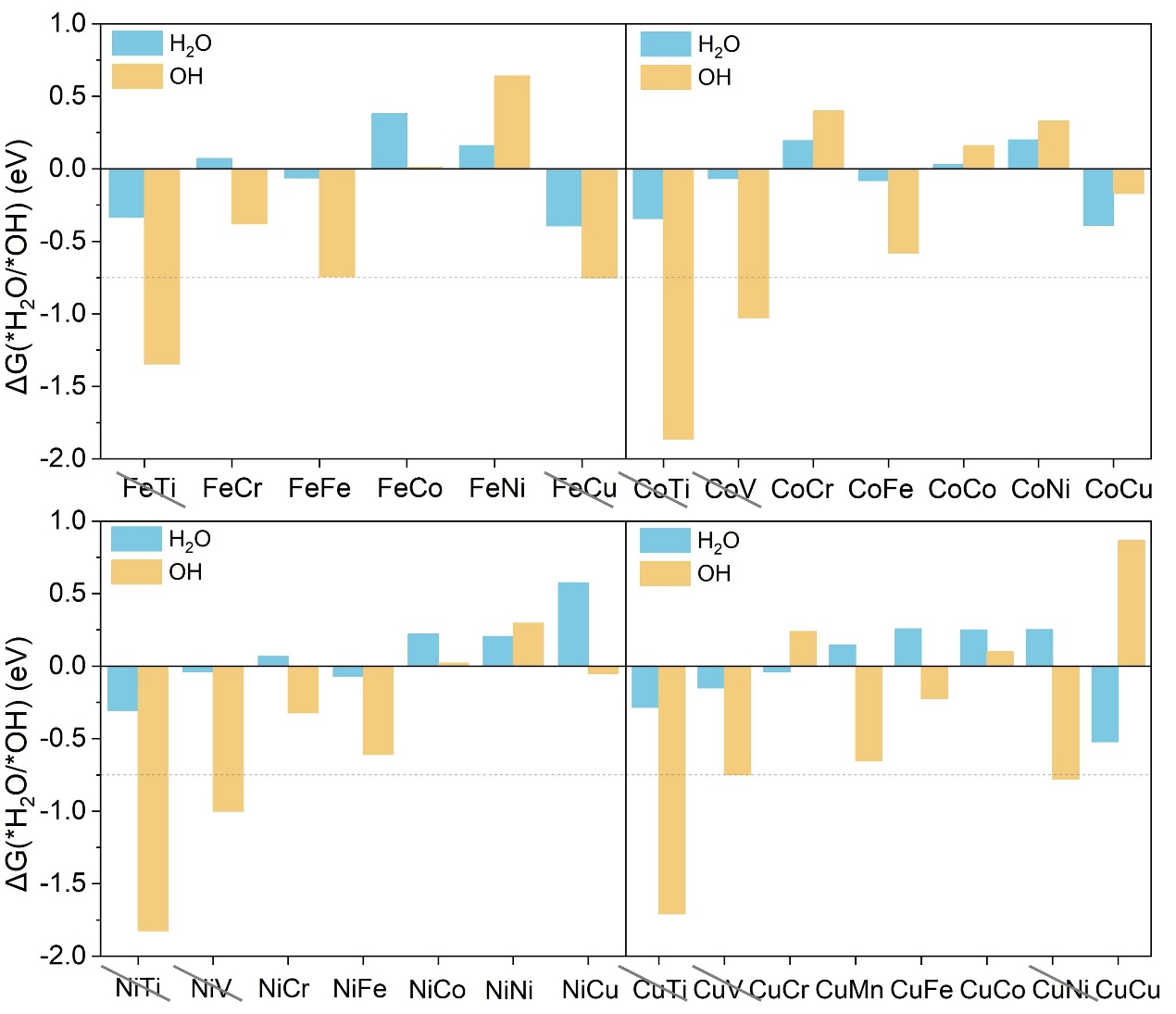


**Figure S3.** The adsorption free energy of OH* and H_2_O on the Janus DACs.


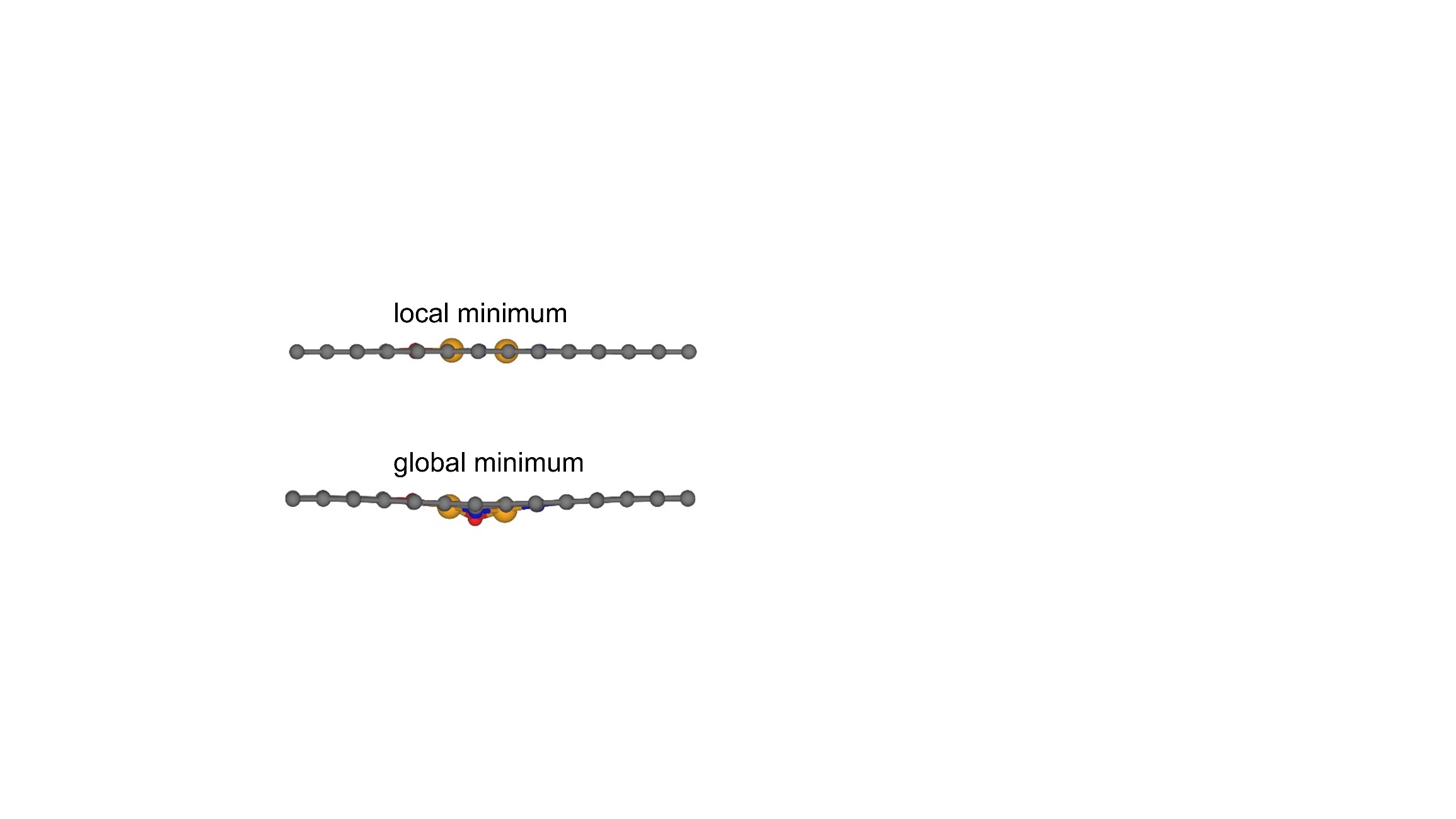


**Figure S4.** The schematic diagram of optimized structure with local minimum and global minimum for some Janus DACs.

**Table S5.** The computed magnetic moment and corresponding total energy of Janus DACs.

| **J-M’M** | **AFM**  **Spin moment**  **(M’ M) (μB)** | **Total Energy**  **(eV)** | **FM**  **Spin moment**  **(M’ M) (μB)** | **Total Energy**  **(eV)** | **NM**  **Spin moment**  **(M’ M) (μB)** | **Total Energy**  **(eV)** |
| --- | --- | --- | --- | --- | --- | --- |
| FeCr | -1.042 2.813 | -631.69 | 1.030 2.557 | -631.62 | - | - |
| FeCo | -1.304 1.448 | -627.96 | 2.088 1.972 | -628.28 | - | - |
| FeFe | -0.045 2.485 | -629.43 | 2.056 3.019 | -629.68 | - | - |
| FeNi | 1.905 -0.546 | -626.8 | - | - | - | - |
| CoCr | - | - | 3.073 0.146 | -630.33 | - | - |
| CoFe | - | - | 0.827 3.073 | -628.27 | - | - |
| CoCo | - | - | 0.546 0.726 | -626.77 | - | - |
| CoNi | -0.677 0.338 | -625.46 | 0.876 1.020 | -625.39 | - | - |
| CoCu | - | - | 0.823 0.129 | -623.58 | - | - |
| NiCr | - | - | 0.236 3.196 | -628.75 | - | - |
| NiFe | - | - | 0.073 2.972 | -626.67 | - | - |
| NiCo | 1.966 -0.039 | -625.2 | - | - | 0 | -625.31 |
| NiNi | - | - | 0.047 0.793 | -624.04 | - | - |
| NiCu | - | - | - | - | 0 | -622.21 |
| CuMn | - | - | 0.009 3.914 | -625.59 | - | - |
| CuFe | - | - | 0.026 3.056 | -624.31 | - | - |
| CuCo | - | - | 0.026 1.919 | -622.77 | - | - |
| CuCu | - | - | 0.235 0.084 | -619.56 | - | - |


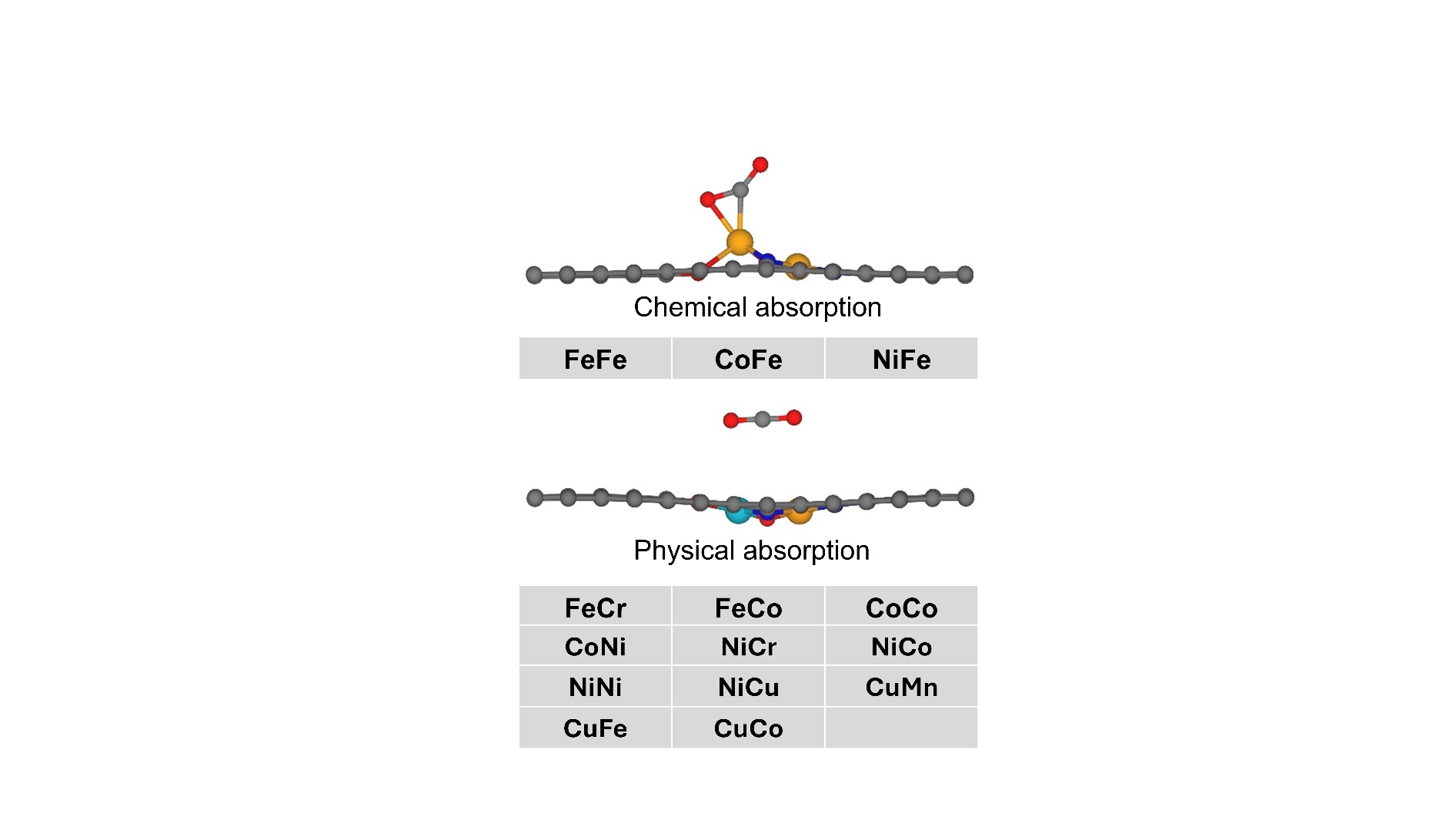


**Figure S5.** The absorption configuration of CO_2_ on Janus DACs.

**Table S6.** The computed CO_2_ adsorption energy of Janus DACs.

| **J-M’M** | **E_ads_ (CO_2_) (eV)** |
| --- | --- |
| FeCr | 0.03 |
| FeCo | 0.08 |
| FeFe | -0.06 |
| CoFe | -0.30 |
| CoCo | 0.09 |
| CoNi | 0.08 |
| NiCr | 0.05 |
| NiFe | -0.34 |
| NiCo | 0.09 |
| NiNi | 0.08 |
| NiCu | 0.05 |
| CuMn | 0.07 |
| CuFe | 0.06 |
| CuCo | 0.06 |


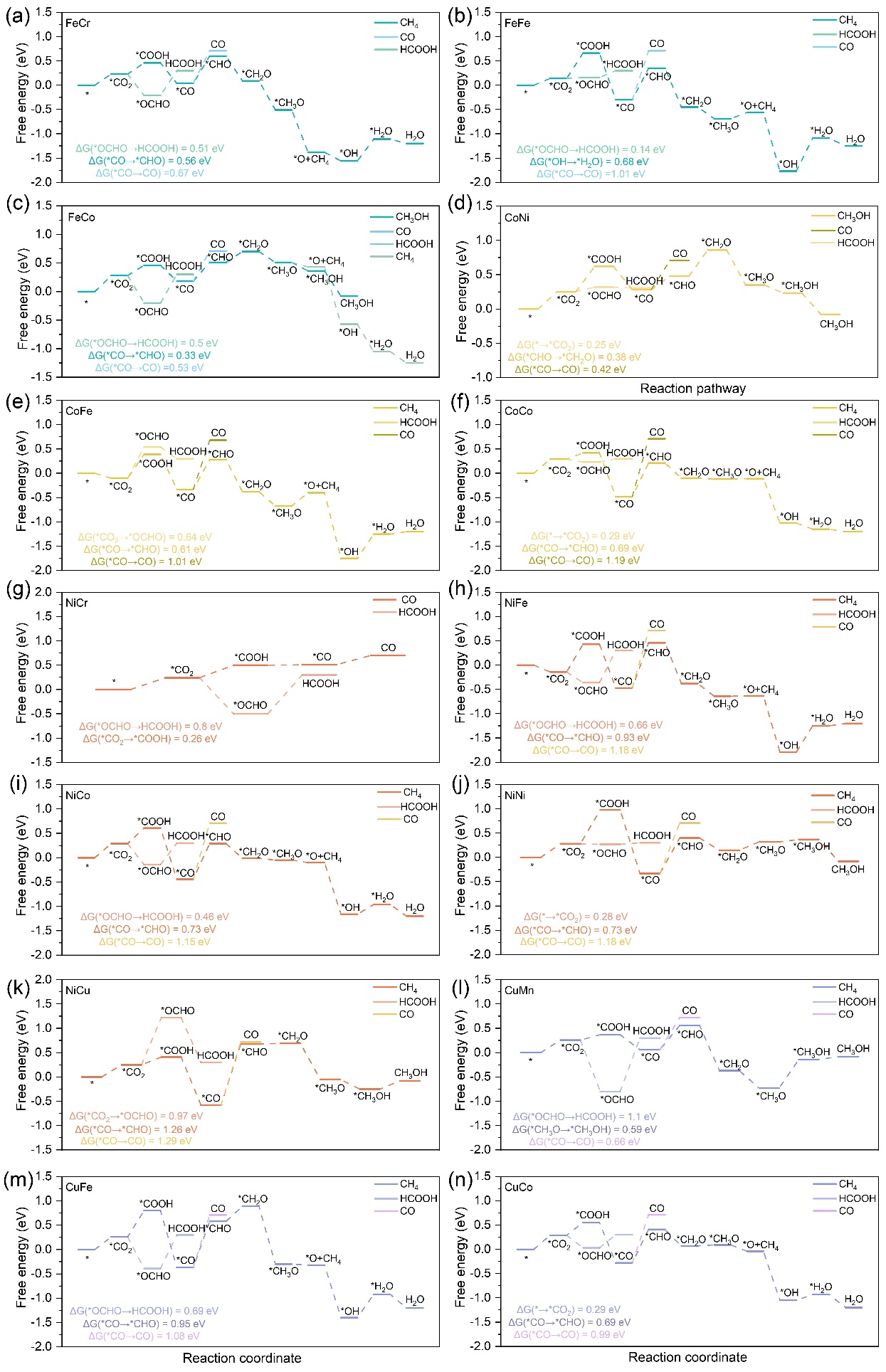


**Figure S6.** The free energy diagram of CO_2_R on (a) J-FeCr, (b) J-FeFe, (c) J-FeCo, (d) J-CoNi, (e) J-CoFe, (f) J-CoCo, (g) J-NiCr, (h) J-NiFe, (i) J-NiCo, (j) J-NiNi, (k) J-NiCu, (l) J-CuMn, (m) J-CuFe, (n) J-CuCo.


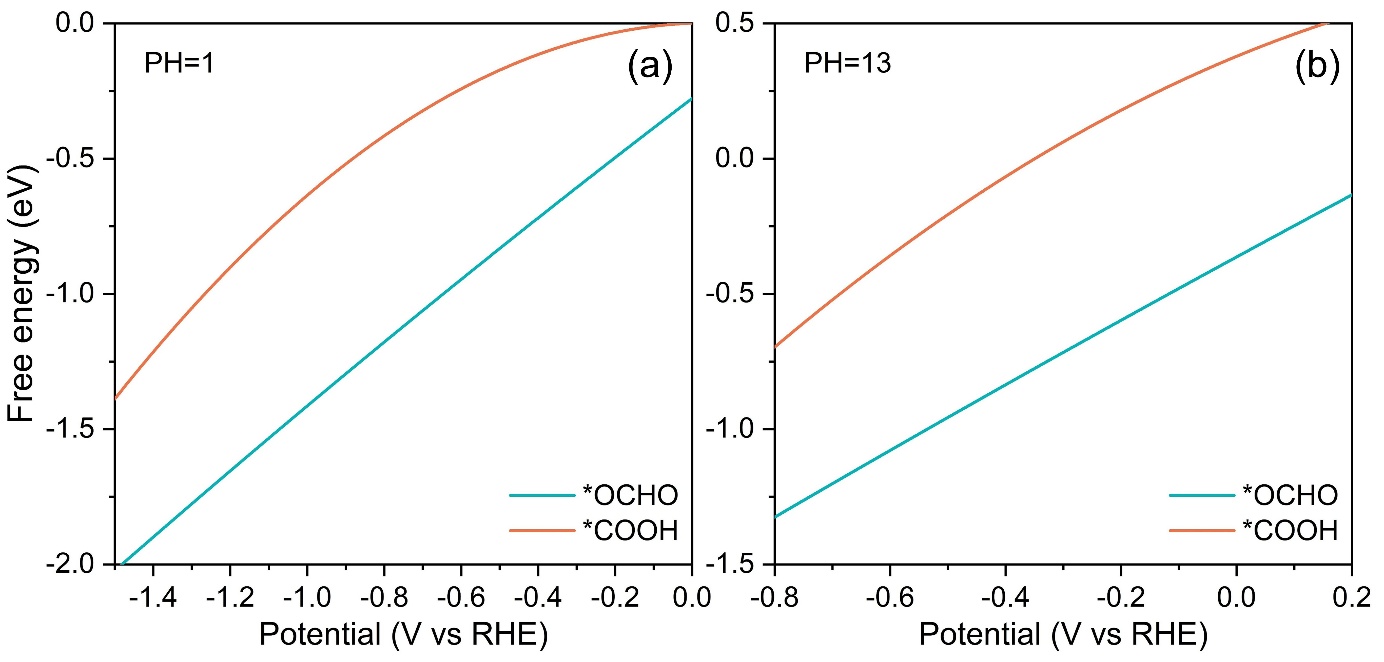


**Figure S7.** The adsorption free energies of *OCHO and *COOH on J-FeCo as a function of potential under (a) PH=1 and (b) PH=13.


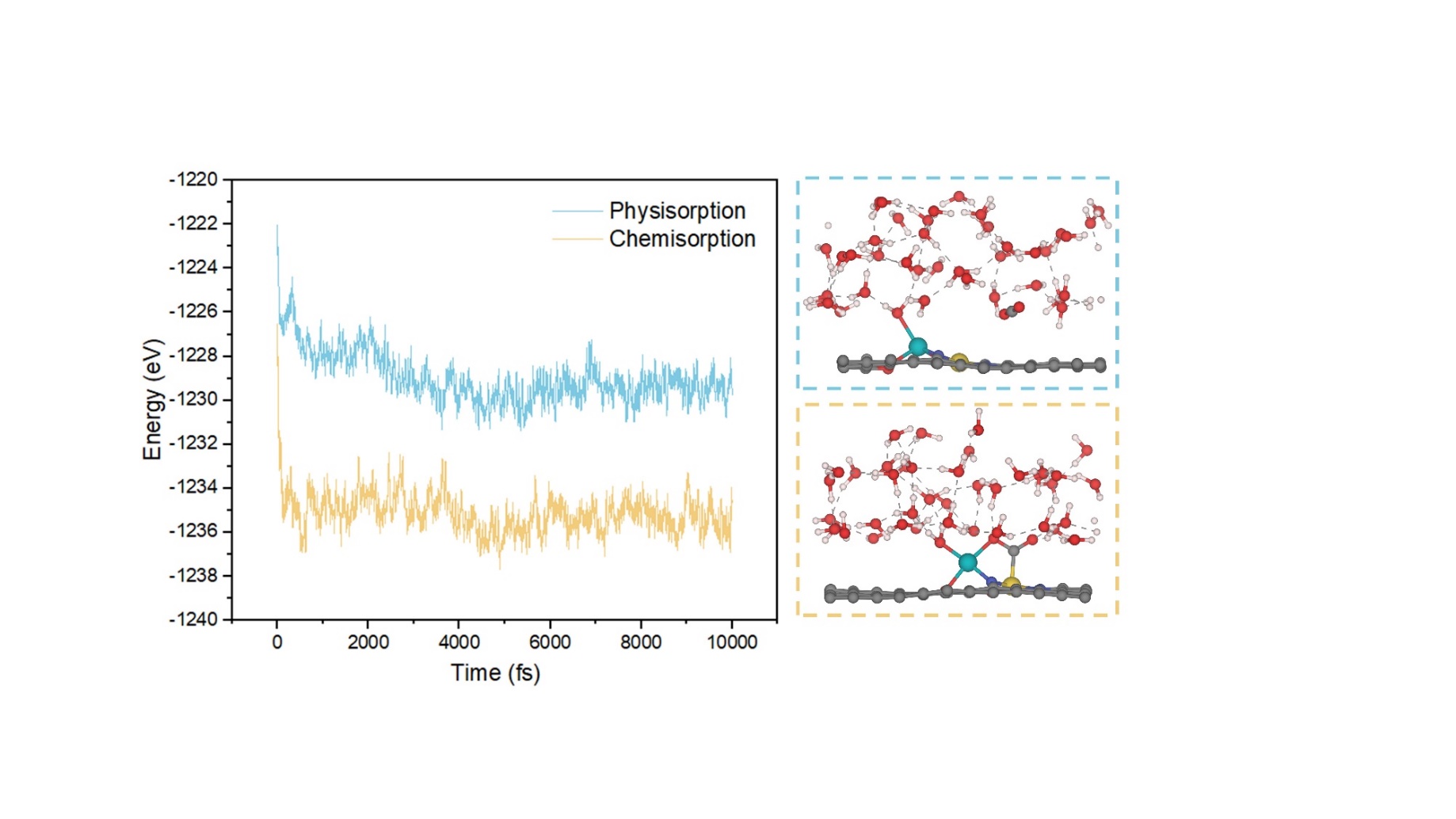


**Figure S8.** The AIMD simulation of CO_2_ adsorption on J-FeCo**.** The snapshots represent the adsorption configuration of CO_2_ which is close to 1ps.

**
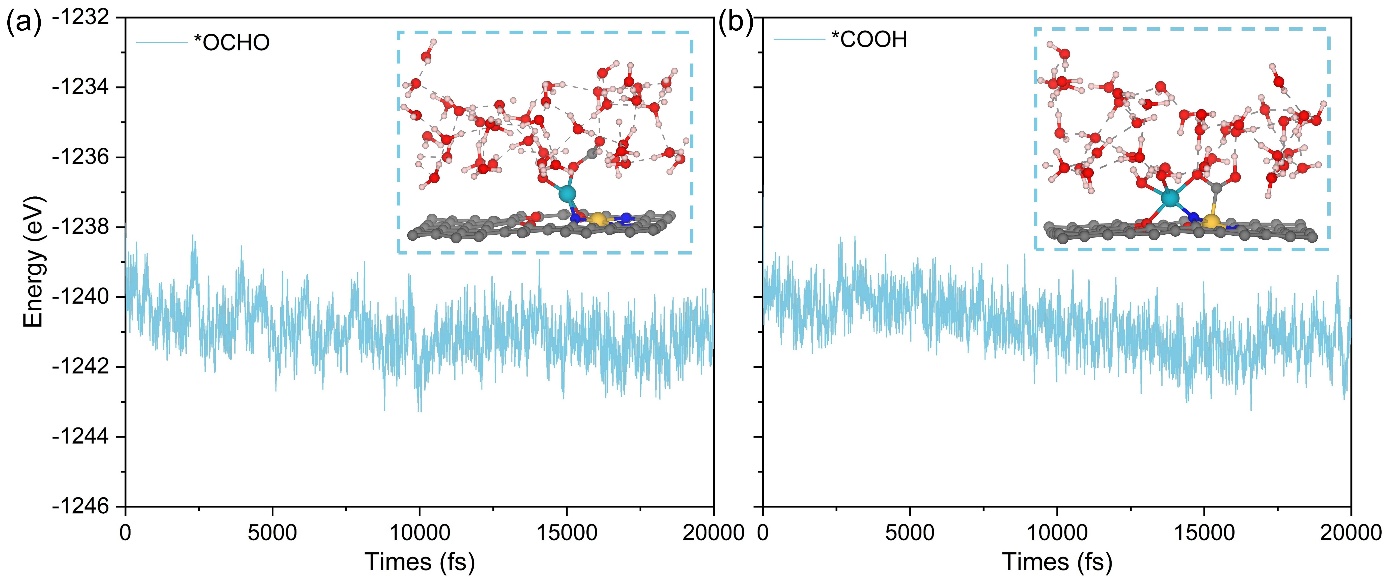
**

**Figure S9.** The AIMD simulation of *COOH and *OCHO adsorptions on J-FeCo**.** The snapshots represent the adsorption configurations of (a) *COOH and (b) *OCHO at 2ps.


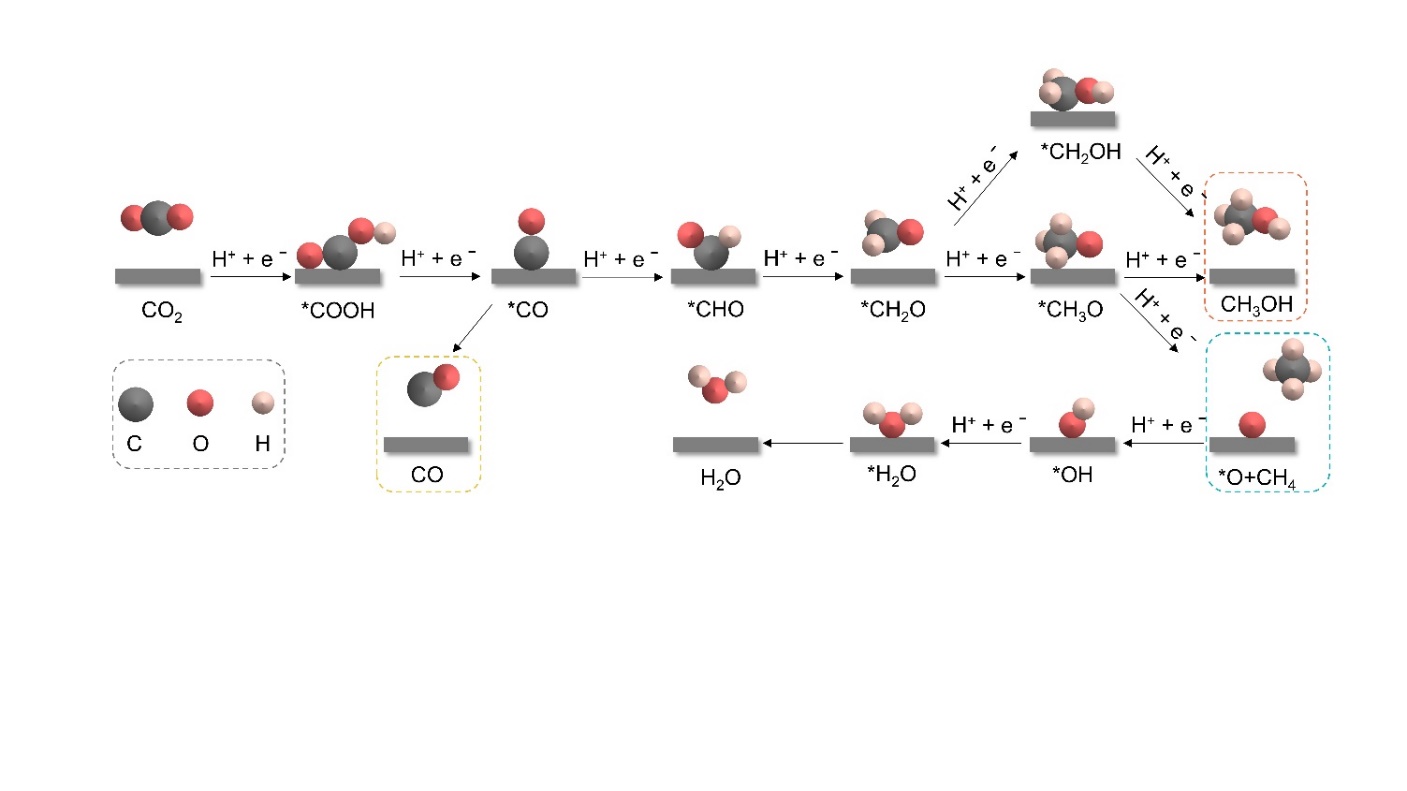


**Figure S10.** The schematic diagram of the reaction pathway of CO_2_R on Janus DACs.


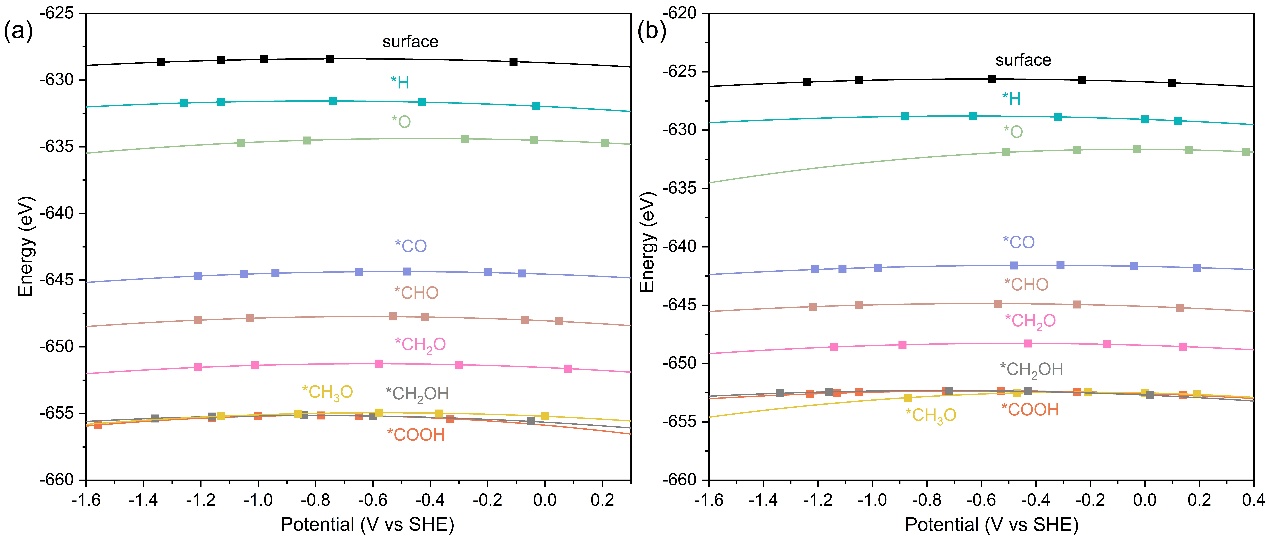


**Figure S11.** The computed total energy of the corresponding reaction intermediates adsorbed on the (a) J-FeCo and (b) J-CoNi as a function of the applied electrode potential.


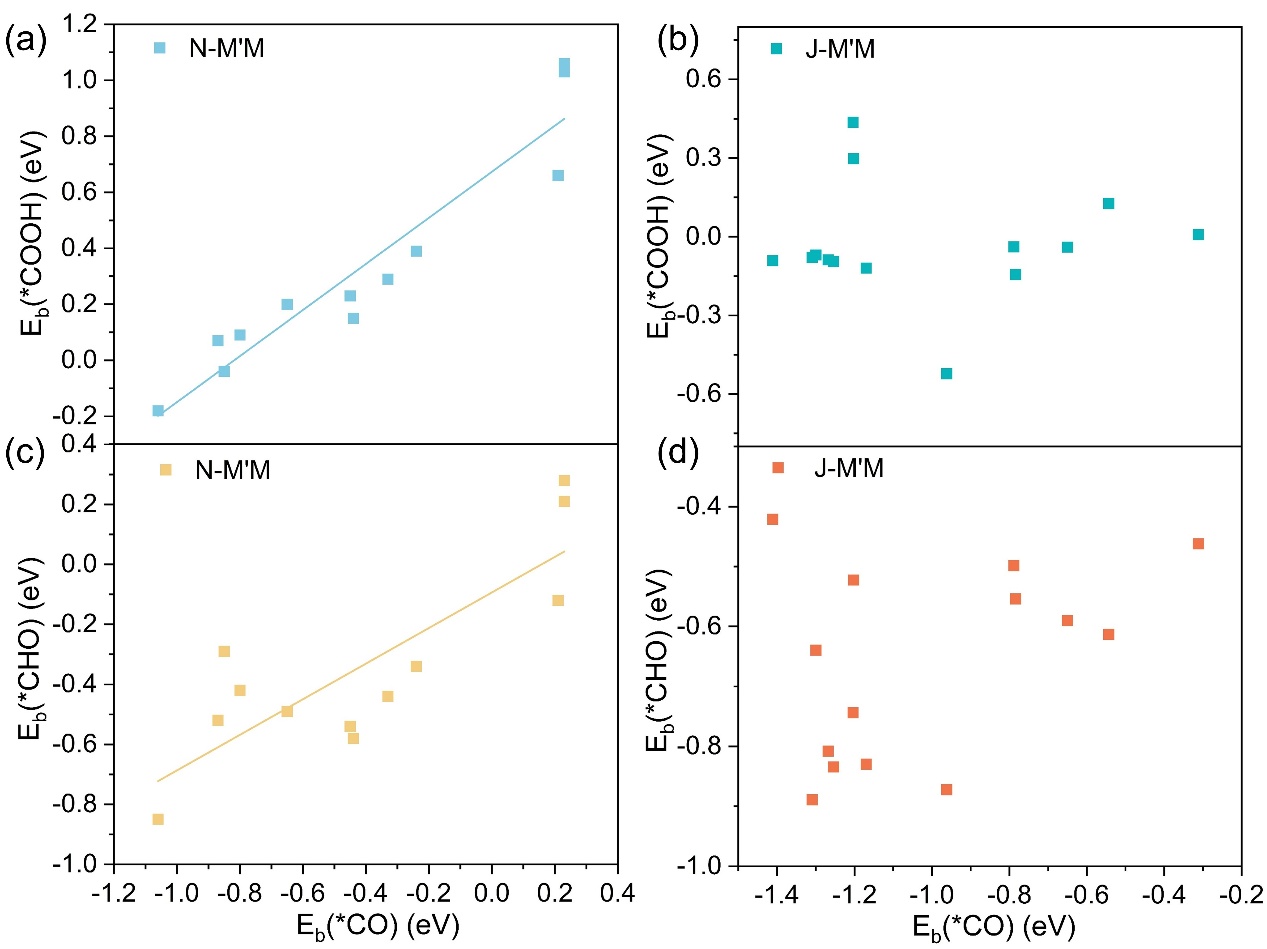


**Figure S12.** The scaling relation between E_b_(*CO) and E_b_(*COOH), E_b_(*CO) and E_b_(*CHO) of (a)(c) normal DACs and (b)(d) Janus DACs.


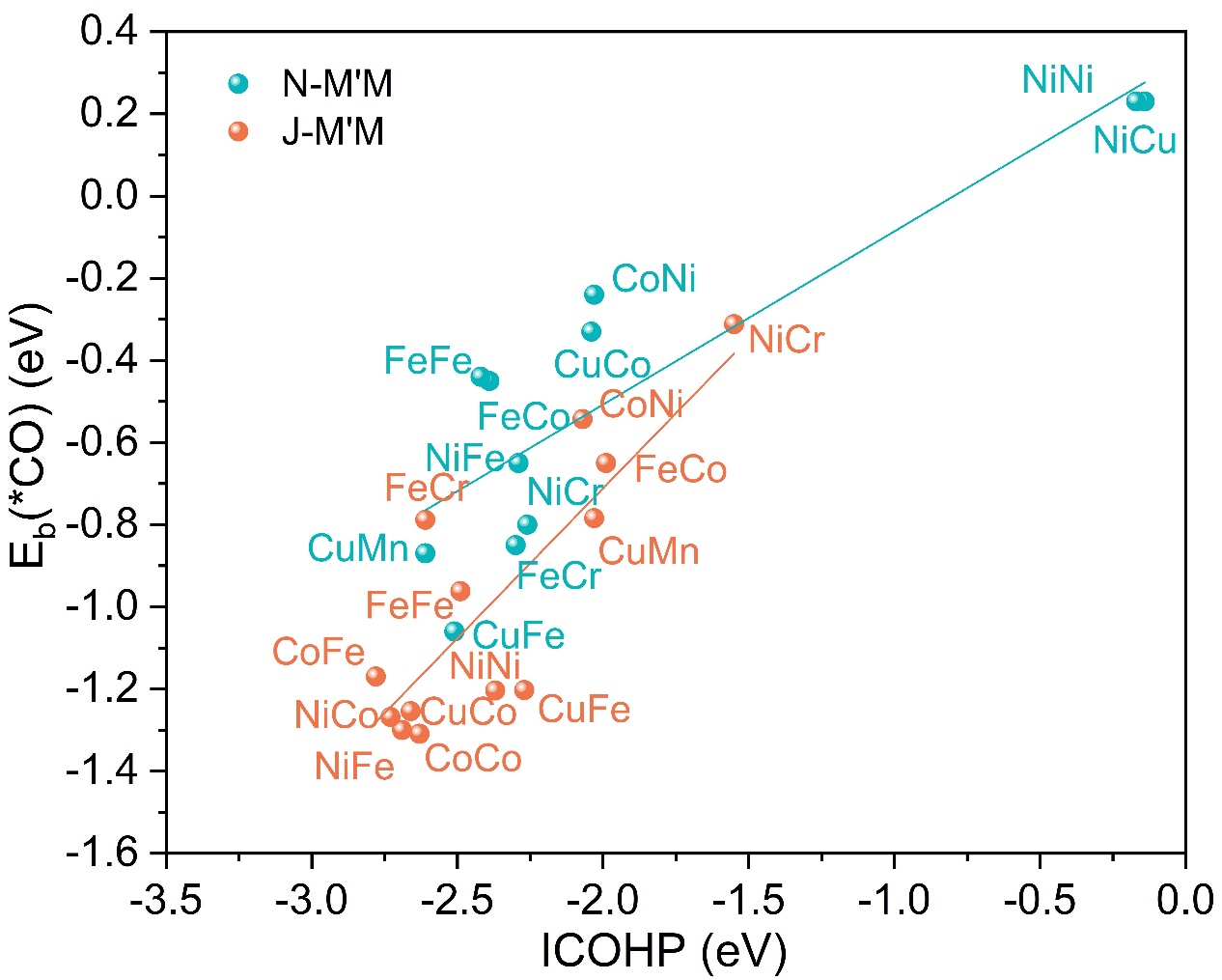


**Figure S13.** The relationship between the computed ICOHP values and the E_b_(*CO) for normal DACs and Janus DACs.


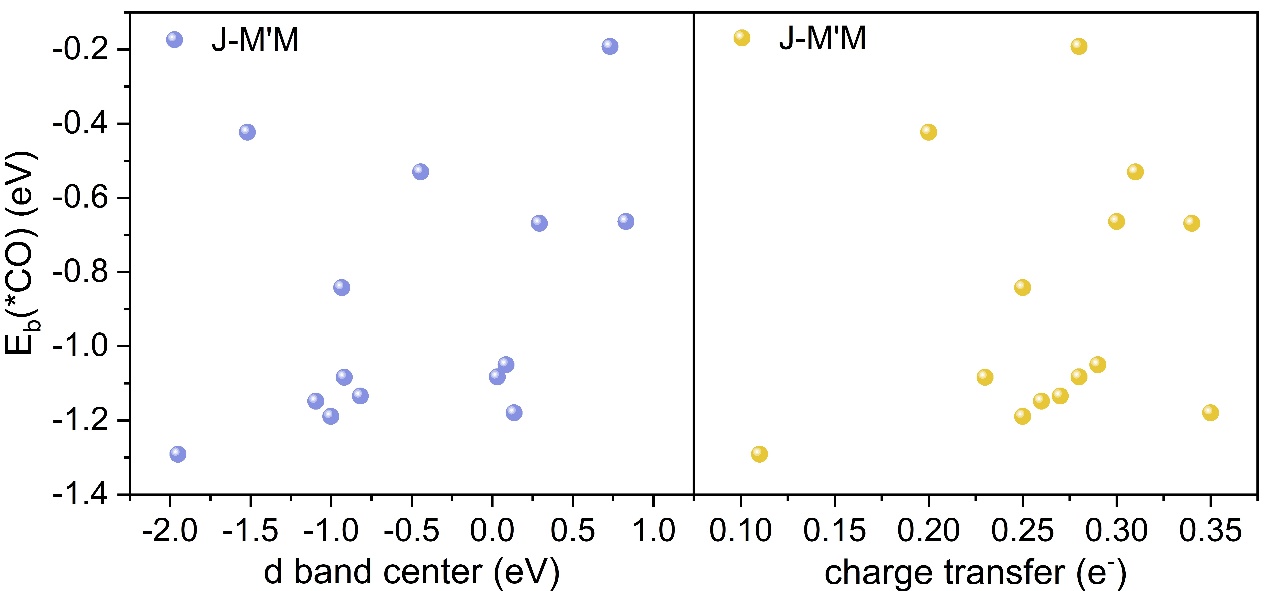


**Figure S14.** The relationship between the values of d band center, charge transfer and E_b_(*CO) on Janus DACs.


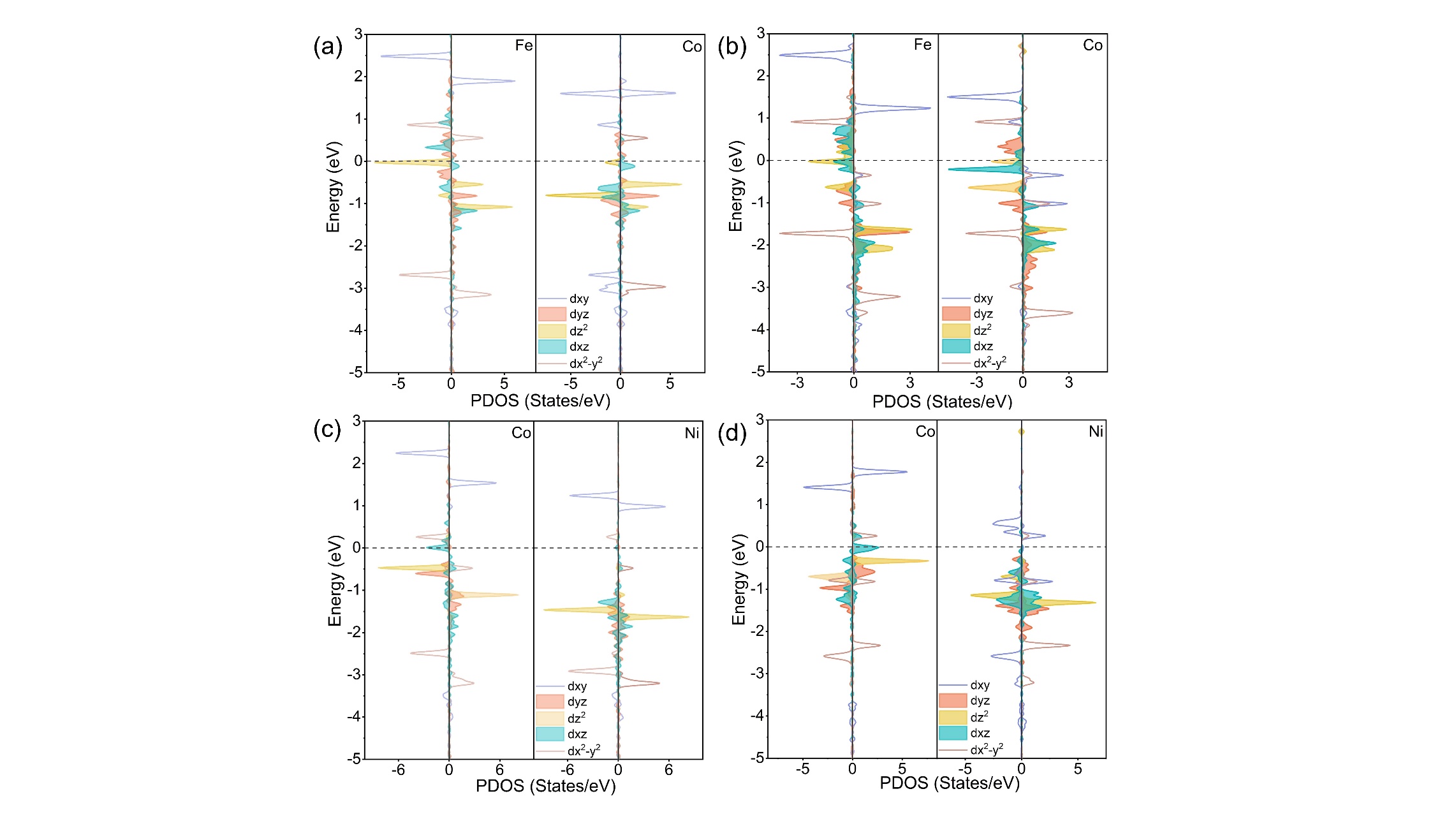


**Figure S15.** Partial density of states (PDOSs) of the Fe, Co-3d and Co, Ni-3d orbitals for (a) N-FeCo, (b) J-FeCo, (c) N-CoNi and (d) J-CoNi. The horizontal dashed lines represent Fermi level.


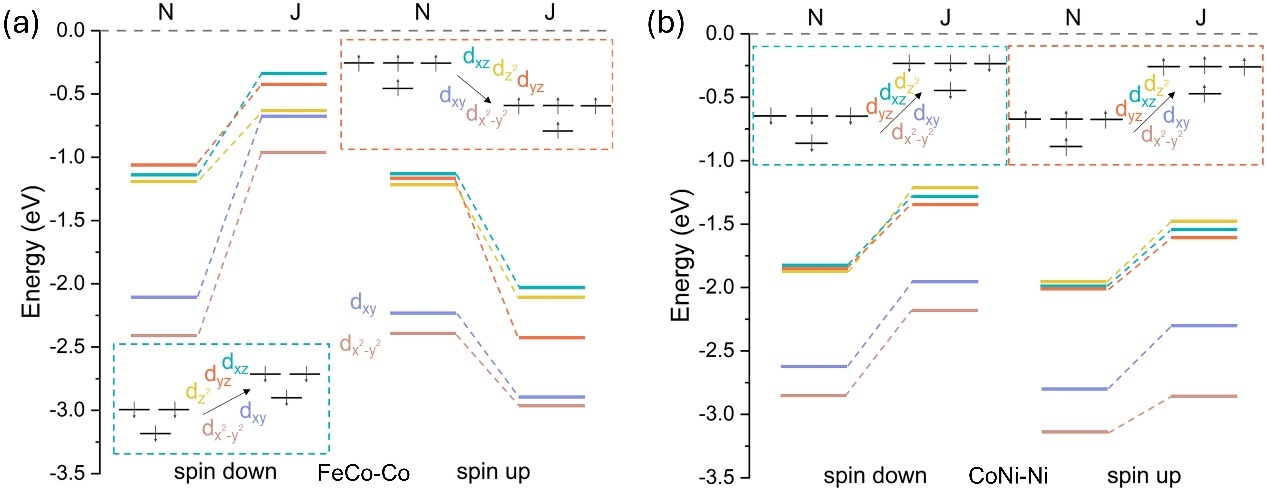


**Figure S16.** The energy level diagrams of 3d orbitals for (a) Co of J-FeCo and N-FeCo, and (b) Ni of J-CoNi and N-CoNi. The insets qualitatively depict the energy levels and electron rearrangement.


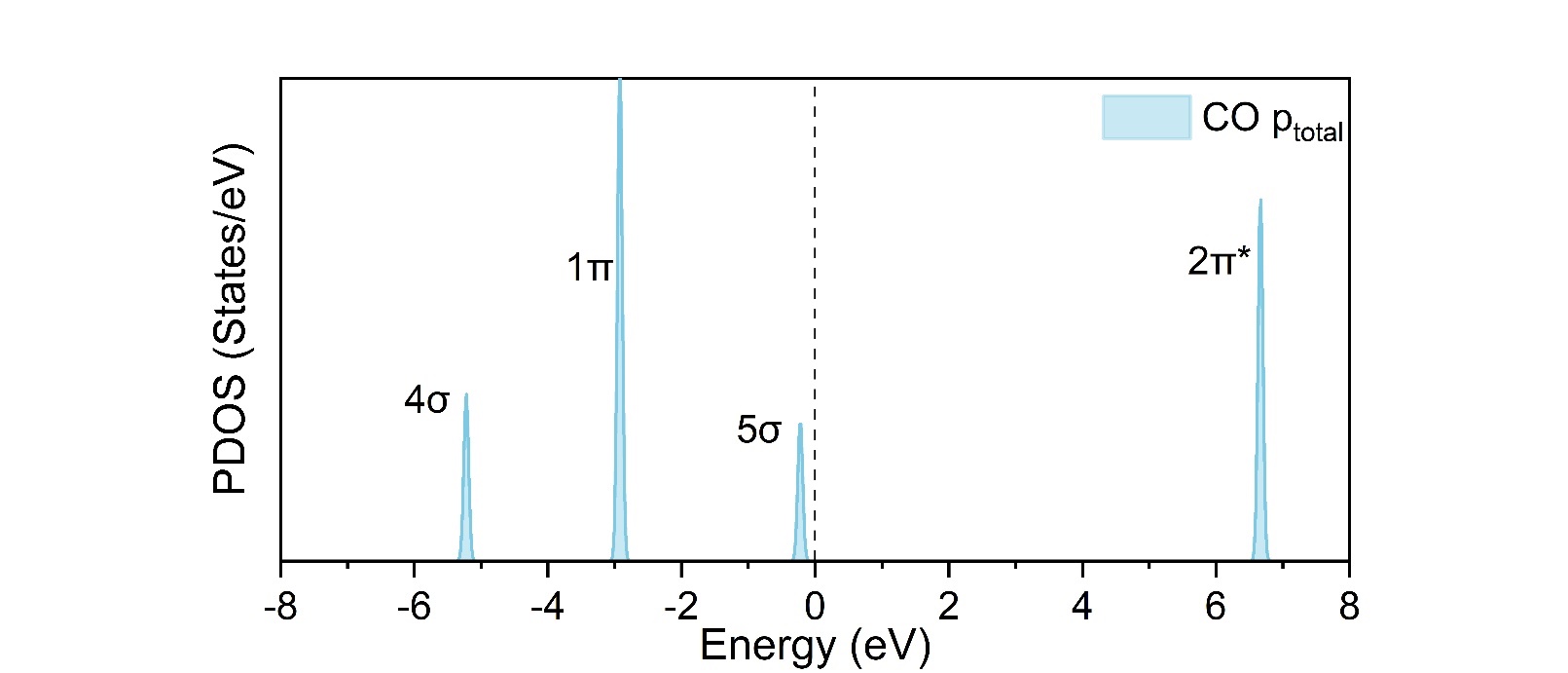


**Figure S17.** PDOSs of 2p orbitals of the CO. The Fermi level is set to zero.


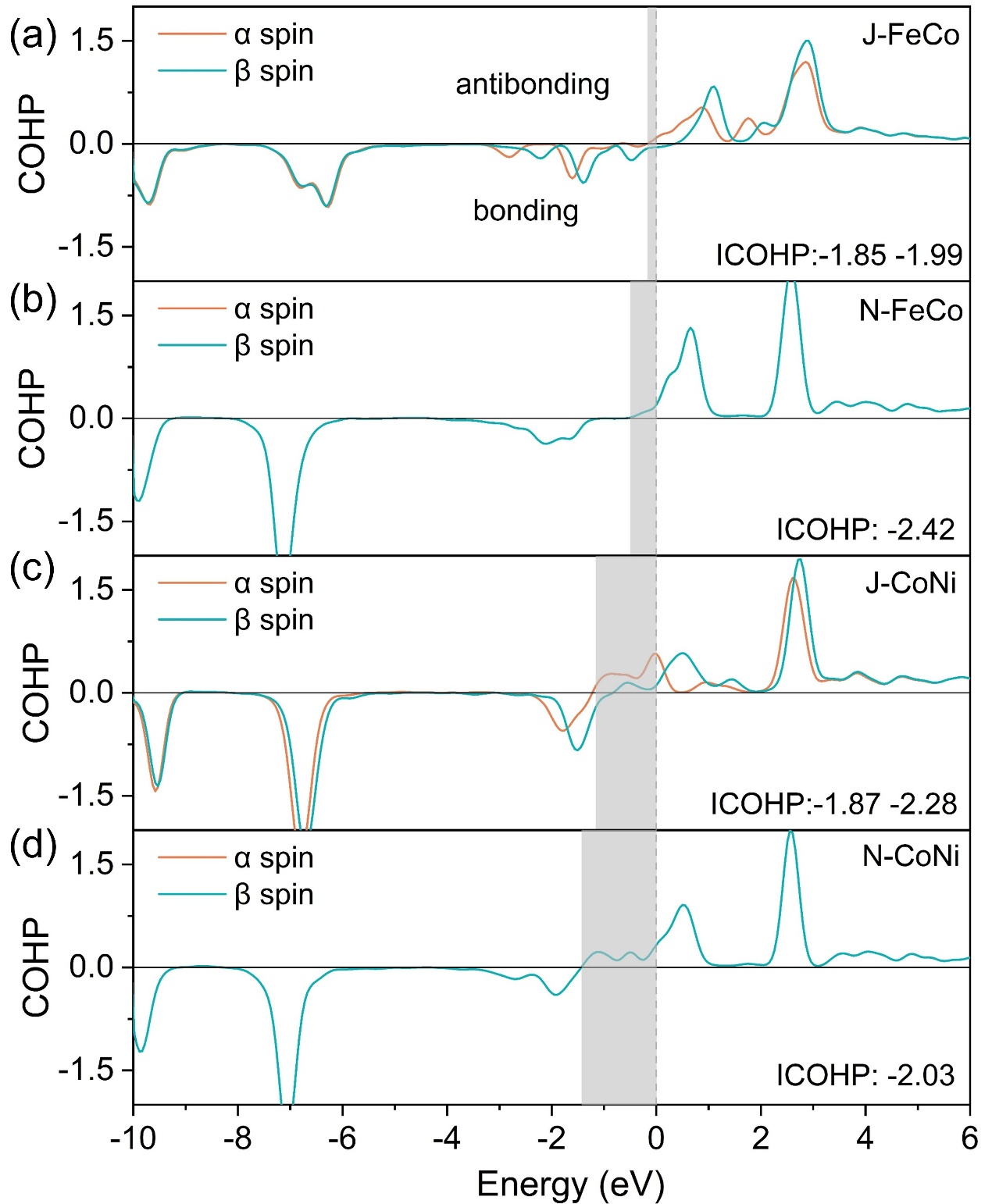


**Figure S18.** COHP between Fe in (a) J-FeCo, (b) N-FeCo and C of adsorbed CO. COHP between Co in (c) J-CoNi, (d) N-CoNi and C of adsorbed CO. Two ICOHP for J- FeCo and J-CoNi values represent α spin and β spin, respectively.

**Table S7.** The values of spin moment for normal DACs and Janus DACs.

| **N-M’M** | **Spin moment**  **M’ (μB)** | **Spin moment**  **M (μB)** | | **J-M’M** | **Spin moment**  **M’ (μB)** | | **Spin moment**  **M (μB)** | | |
| --- | --- | --- | --- | --- | --- | --- | --- | --- | --- |
| FeCr | 1.099 | | 1.796 | FeCr | | 1.03 | | 2.557 |  |
| FeFe | 1.128 | | 1.129 | FeFe | | 2.056 | | 3.019 |  |
| FeCo | -0.004 | | 1.153 | FeCo | | 2.088 | | 1.972 |  |
| CoCo | 0 | | 0 | CoFe | | 0.827 | | 3.073 |  |
| CoNi | -0.95 | | -0.185 | CoCo | | 0.546 | | 0.726 |  |
| NiCr | -0.013 | | 2.339 | CoNi | | -0.677 | | 0.338 |  |
| NiFe | 0.166 | | 2.224 | NiCr | | 0.236 | | 3.196 |  |
| NiNi | 0 | | 0 | NiFe | | -0.073 | | 2.972 |  |
| NiCu | 0 | | 0 | NiCo | | 0 | | 0 |  |
| CuMn | -0.269 | | 3.029 | NiNi | | 0.047 | | 0.793 |  |
| CuFe | 0.44 | | 0.053 | NiCu | | 0 | | 0 |  |
| CuCo | 0.53 | | 1 | CuMn | | 0.009 | | 3.914 |  |
|  |  | |  | CuFe | | 0.026 | | 3.056 |  |
|  |  | |  | CuCo | | 0.026 | | 1.919 |  |


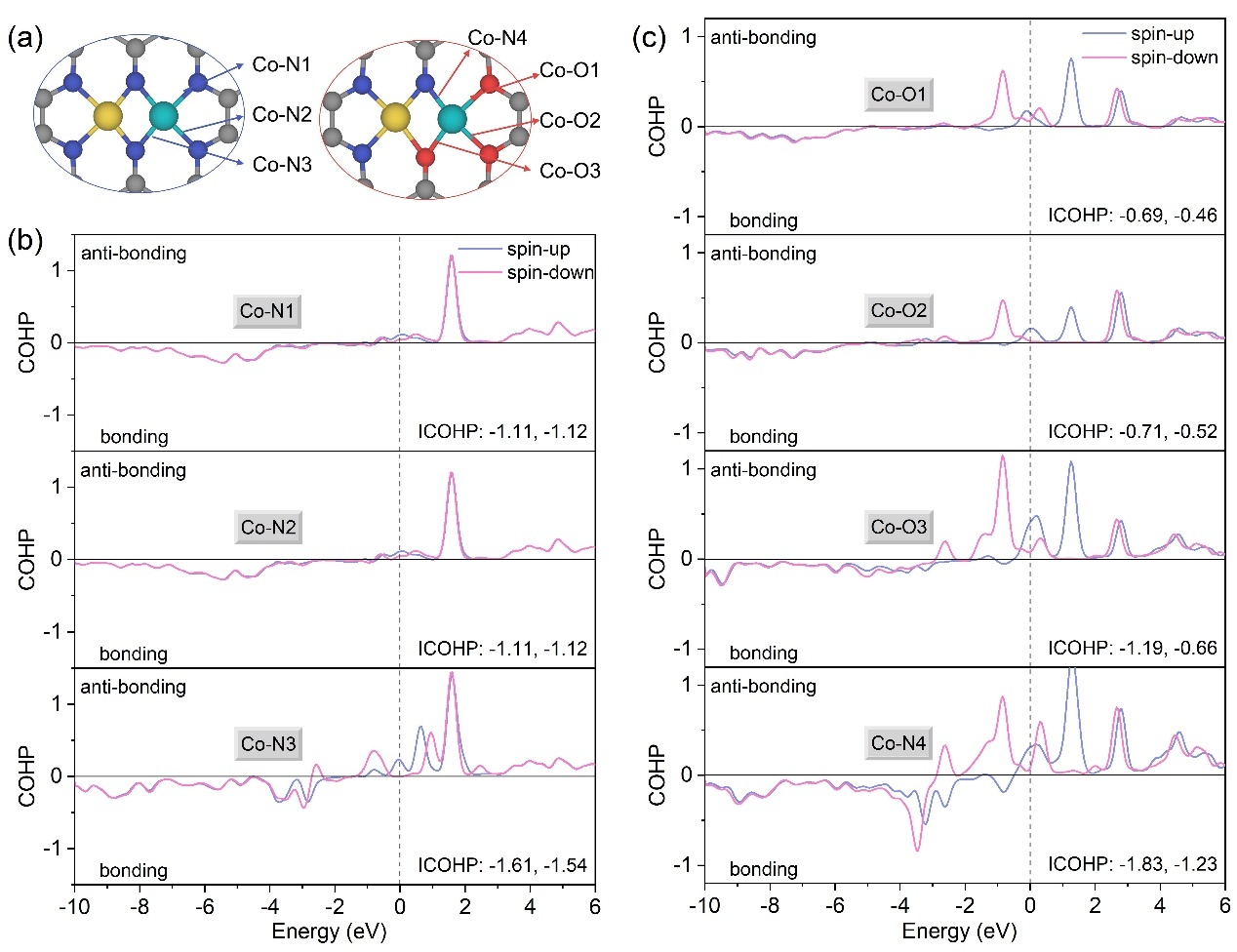


**Figure S19.** (a) The schematic of labels of bonding between Co and N, O atoms for N-FeCo and J-FeCo DACs. COHP between the central Co atom and its coordination N atoms and O atoms for (b) N-FeCo (c) J-FeCo DACs, respectively. Two ICOHP values represent spin-up and spin-down, respectively.

**
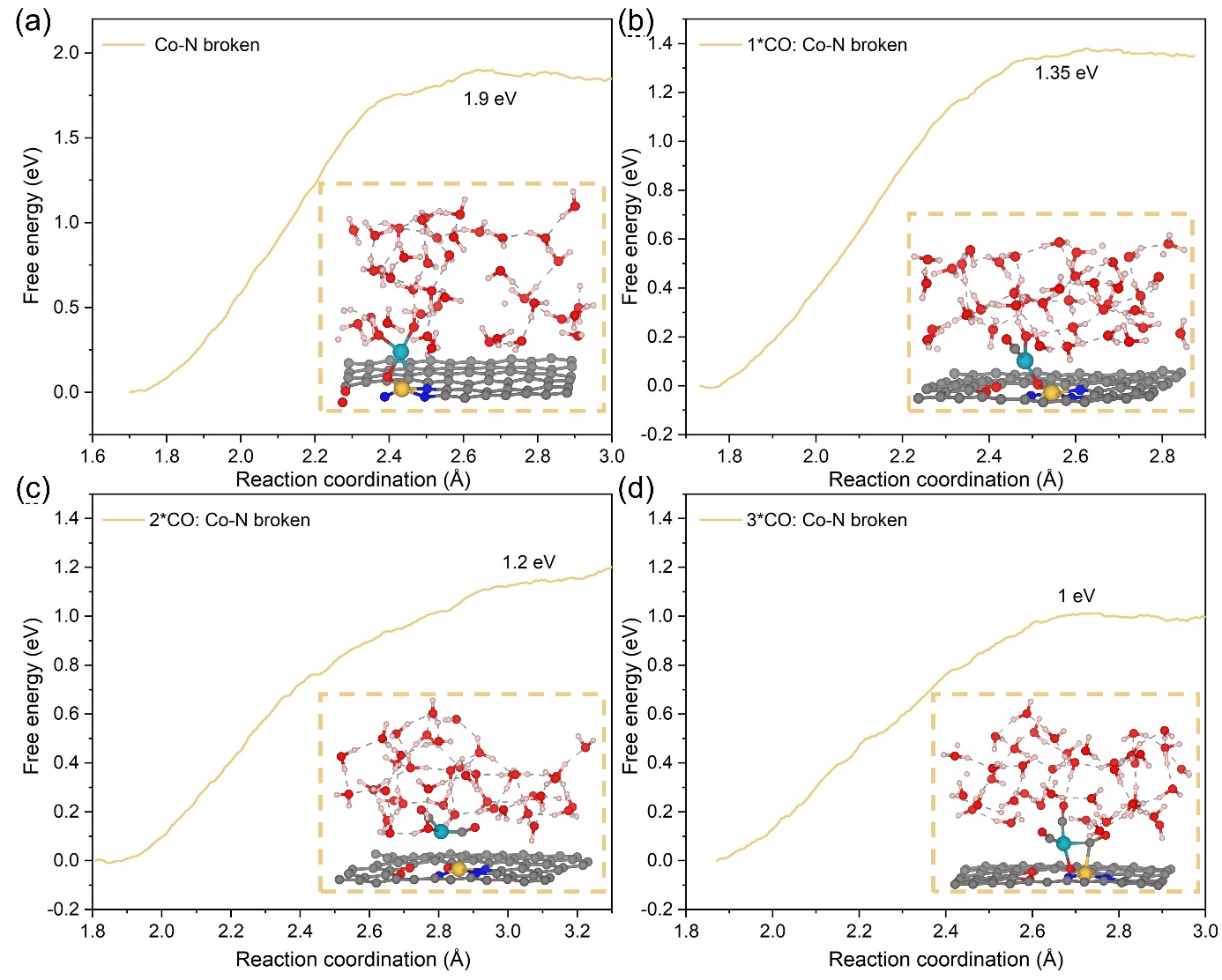
**

**Figure S20.** The kinetic barrier for Co-N bond cleavage (a) under solvent environment and (b-c) across varying CO coverages.

**Table S8.** The collection of the values of the property and features for Janus DACs on both sites.

| **J-M’M-active site** | **E_b_(*CO) (eV)** | **d electron number** | **χ_M’/M_/χ_O/N_** | **χ_M/M’_/χ_N/O_** |
| --- | --- | --- | --- | --- |
| FeTi-Fe | -0.47 | 6 | 0.601973 | 0.447674 |
| FeTi-Ti | -0.67 | 2 | 0.447674 | 0.601973 |
| FeCr-Cr | -0.7884 | 5 | 1.453488 | 0.601974 |
| FeCr-Fe | -0.7084 | 6 | 0.601974 | 1.453488 |
| FeFe-Fe2 | -0.9621 | 6 | 0.531977 | 0.601974 |
| FeFe-Fe1 | -1.1321 | 6 | 0.601974 | 0.531977 |
| FeCo-Co | -0.65 | 6 | 0.601974 | 0.546512 |
| FeCo-Fe | -0.62 | 7 | 0.546512 | 0.601974 |
| FeNi-Fe | -0.46 | 6 | 0.601973 | 0.555232 |
| FeNi-Fe | -1.12 | 8 | 0.555232 | 0.601973 |
| FeCu-Fe | -0.53 | 6 | 0.601973 | 0.552325 |
| FeCu-Cu | -1.19 | 10 | 0.552325 | 0.601973 |
| CoTi-Ti | -0.89 | 2 | 0.447674 | 0.618421 |
| CoV-Co | -0.42 | 7 | 0.618421 | 0.473837 |
| CoV-V | -0.92 | 3 | 0.473837 | 0.618421 |
| CoCr-Co | -0.48 | 7 | 0.618421 | 1.453488 |
| CoCr-Cr | -0.68 | 5 | 1.453488 | 0.618421 |
| CoFe-Fe | -1.1699 | 6 | 0.531977 | 0.618421 |
| CoFe-Co | -0.7599 | 7 | 0.618421 | 0.531977 |
| CoCo-Co2 | -1.3091 | 7 | 0.546512 | 0.618421 |
| CoCo-Co1 | -0.5991 | 7 | 0.618421 | 0.546512 |
| CoNi-Co | -0.5433 | 7 | 0.618421 | 0.555233 |
| CoNi-Ni | -1.2533 | 8 | 0.555233 | 0.618421 |
| CoCu-Co | -0.64 | 7 | 0.618421 | 0.552325 |
| CoCu-Cu | -1.21 | 10 | 0.552325 | 0.618421 |
| NiTi-Ni | 0 | 8 | 0.628289 | 0.447674 |
| NiTi-Ti | -0.88 | 2 | 0.447674 | 0.628289 |
| NiV-Ni | -0.3 | 8 | 0.628289 | 0.473837 |
| NiV-V | -0.86 | 3 | 0.473837 | 0.628289 |
| NiCr-Cr | -0.312 | 5 | 1.453488 | 0.628289 |
| NiCr-Ni | -0.362 | 8 | 0.628289 | 1.453488 |
| NiFe-Fe | -1.2997 | 6 | 0.531977 | 0.628289 |
| NiFe-Ni | -0.3197 | 8 | 0.628289 | 0.531977 |
| NiCo-Co | -1.2681 | 7 | 0.546512 | 0.628289 |
| NiCo-Ni | -0.0881 | 8 | 0.628289 | 0.546512 |
| NiNi-Ni2 | -1.2038 | 8 | 0.555233 | 0.628289 |
| NiNi-Ni1 | 0.1262 | 8 | 0.628289 | 0.555233 |
| NiCu-Cu | -1.4112 | 10 | 0.552326 | 0.628289 |
| NiCu-Ni | 0.0088 | 8 | 0.628289 | 0.552326 |
| CuTi-Ti | -0.88 | 2 | 0.447674 | 0.625 |
| CuV-V | -0.82 | 3 | 0.473837 | 0.625 |
| CuCr-Cu | -0.3 | 10 | 0.625 | 1.453488 |
| CuCr-Cr | -0.64 | 5 | 1.453488 | 0.625 |
| CuMn-Mn | -0.784 | 5 | 0.450581 | 0.625 |
| CuMn-Cu | -0.414 | 10 | 0.625 | 0.450581 |
| CuFe-Fe | -1.2025 | 6 | 0.531977 | 0.625 |
| CuFe-Cu | -0.3625 | 10 | 0.625 | 0.531977 |
| CuCo-Co | -1.2543 | 7 | 0.546512 | 0.625 |
| CuCo-Cu | -0.4643 | 10 | 0.625 | 0.546512 |
| CuNi-Cu | 0.03 | 10 | 0.625 | 0.555232 |
| CuNi-Ni | -1.06 | 8 | 0.555232 | 0.625 |
| CuCu-Cu | -1.21 | 10 | 0.552325 | 0.625 |

**References**

[1] T. K. Woo, P. M. Margl, P. E. Blo1chl, T. A. Ziegler, *J. Phys. Chem. B.* **1997**, 101, 7878-7880.

[2] C. Braga, K. P. Travis, *J. Chem. Phys*. **2005**, *123*, 134101.

[3] X. Zhao, J. Shi, Y. Ji, Y. Liu, *WIREs Comput. Mol. Sci*. **2019**, *9*, e1418.

[4] D. Kim, J. Shi, Y. Liu, *J Am Chem Soc*. **2018**, *140*, 9127-9131.

[5] K. Mathew, V. S. C. Kolluru, S. Mula, S. N. Steinmann, R. G. Hennig, *J. Chem. Phys*. **2019**, *151*, 234101.

[6] J. C. Liu, F. Luo, J. Li, *J. Am. Chem. Soc*. **2023**, *145*, 25264-25273.

[7] R. Ouyang, S. Curtarolo, E. Ahmetcik, M. Scheffler, L. M. Ghiringhelli, *Phys. Rev. Mater*. **2018**, *2*, 083802.

[8] X. Guo, J. Gu, S. Lin, S. Zhang, Z. Chen, S. Huang, *J. Am. Chem. Soc*. **2020**, *142*, 5709-5721.
